# Climate change drives persistent organic pollutant dynamics in marine environments

**DOI:** 10.1038/s43247-025-02348-4

**Published:** 2025-05-13

**Authors:** Pamela D. Noyes, Daniele Miranda, Gabriel Oliveira de Carvalho, Alessandra Perfetti-Bolaño, Yago Guida, Fábio Barbosa Machado Torres, João Paulo Machado Torres, Karina S. B. Miglioranza, Vanessa Hatje, Ricardo O. Barra

**Affiliations:** 1 Integrated Climate Sciences Division, Center for Public Health and Environmental Assessment, Office of Research and Development, U.S. EPA, Washington, DC, USA.; 2 University of Notre Dame, Department of Biological Sciences, Notre Dame, IN, USA.; 3 Instituto de Biofísica Carlos Chagas Filho, Centro de Ciências da Saúde, Universidade Federal do Rio de Janeiro, Ilha do Fundão, Rio Janeiro, RJ, Brazil.; 4 Facultad de Ciencias Ambientales y Centro EULA, Centro FONDAP CRHIAM, Universidad de Concepción, Concepción, Chile.; 5 Universidad Nacional de Mar del Plata, IIMyC (CONICET), Mar del Plata, Argentina.; 6 Centro Interdisciplinar de Energia e Ambiente (CIEnAm) & Department of Analytical Chemistry, Universidade Federal da Bahia, Salvador, Brazil.; 7 Marine Environmental Studies Laboratory, IAEA Marine Environment Laboratories, Department of Nuclear Sciences and Applications, International Atomic Agency, Monaco, Monaco.; 8 Instituto Milenio de Socio-Ecología Costera (SECOS), Concepción, Chile.

## Abstract

Understanding climate change impacts in combination with other anthropogenic stressors, such as chemical pollution, is critical to identifying vulnerable marine ecosystems. This paper presents a systematic review and conceptual model mapping evidence of the marine environmental fate and biological effects of persistent organic pollutants with shifting climate drivers. Increasing ice melt, atmospheric deposition, and sediment remobilization are altering persistent organic pollutant dynamics in northern polar environments, but with data gaps elsewhere. While limited to fish and invertebrates, principal biological effect pathways involve reduced survival and perturbed thermal regulation and bioenergetics, notably in some populations residing in more heavily polluted and thermal edge habitats. Associated food web shifts with climate change are also altering persistent organic pollutant bioaccumulation among some marine mammal and seabird populations and assemblages. The evidence suggests potential ecological deterioration in some areas, with many unknowns underscoring the need for advancing experimental and modeling tools to evaluate these complex interactions.

The physicochemical properties of the ocean environment are changing (e.g., warming, acidification, deoxygenation), leading to more frequent and intense marine heatwaves, glacial retreat and cryosphere melting, and sea level rise (see IPCC findings TS.2.4 to TS.2.6)^1,2^. In its most recent assessment, the Intergovernmental Panel on Climate Change (IPCC) describes the role of climate-driven shifts in altering and deteriorating ecosystem structure and function, and reducing the resilience and adaptive capacities of species, populations, and community assemblages (e.g., see IPCC key finding TS.B.1)^3^. Additionally, the IPCC has projected that cumulative stressors and extreme events will increase in magnitude and frequency, exacerbating stress on ecosystem integrity (e.g., see IPCC key finding TS.C.2)^3^. One of the continuing less understood aspects of these multi-stressor impacts relates to its interactions with and influence on the environmental fate and biological effects of persistent organic pollutants (POPs; see Table 1)^4,5^.

POPs represent a diverse class of mostly man-made carbon-based chemicals with large scale historical and current uses as pesticides and in industrial applications and consumer products, as well as being unintentional byproducts of manufacturing and waste incineration^6^. The Stockholm Convention, adopted in 2001 and implemented in 2004, is a global treaty managed by the United Nations Environment Programme with goals of eliminating and reducing POP production, trading, and use. The POP Global Monitoring Program under the Stockholm Convention collects and monitors POP environmental levels with use restrictions^7^. Twelve chemicals (i.e., legacy POPs) were the initial focus of the Convention with chemicals being added periodically based on persistence, bioaccumulation, toxicity, and long-range transport potential^5^).

The properties of POPs that make them a priority concern for human and environmental health have been described at length under the United Nation (UN) Stockholm Convention^6,8–14^. POPs are highly persistent and bioaccumulative in humans and wildlife with extensive global distribution due to their widespread use and intrinsic physiochemical properties. POPs may be deposited to marine environments through atmospheric deposition, effluent releases, and land surface runoff. To varying degrees, POPs are resistant to environmental breakdown by photolytic, chemical, and biological processes, and may occur in water in dissolved phases or adsorbed to particles, and likewise in the atmosphere in vapor or particulate phases^6,9–11^. The persistence and semi-volatility of POPs makes them prone to long-range atmospheric and oceanic transport, in some cases far from their initial source^14,15^. The generally low water solubility of POPs also facilitates adsorption to particulate and organic matter in soils and sediments that can act as long-term reservoirs with re-entry into global circulation when these areas are disturbed^9^. POPs tend to be highly fat soluble, readily sequestering to lipid rich tissues of exposed organisms, allowing for their accumulation in biota^16–18^. Exceptions to lipid accumulation include the listed per- and polyfluoroalkyl substances (PFAS) chemicals that tend to bind to serum proteins^19^.

The importance of understanding the potential consequences of climate drivers on POP exposure and health effect pathways is well-recognized^7–9,20–27^. For example, the Arctic Monitoring and Assessment Programme (AMAP) has assessed POP trends in Arctic environments for many years, including efforts to evaluate chemical trends with climate change (see Supplementary Table 2). More recent focus has expanded to climate shifts in POP temporal trends in the Southern Hemisphere and Antarctica^28–30^.

One challenge in understanding the consequences of the climate-POP nexus on the marine environment is the extensive biogeochemical and spatiotemporal variability of thermally, structurally, and biologically diverse ecosystems represented by coastal zones, estuaries, coral reefs, saltwater marshes, and mangrove forests, as well as open and deep-sea oceanic systems, among many others. At a conceptual level, climate change may impact the capacity of species and populations to respond and adapt to POP exposures (and other anthropogenic stressors). Climate change thus can be an environmental driver increasing chemical bioavailability and toxicological effects that may propagate to degraded community and ecosystem structure, health, and resilience. Reciprocally, ongoing and new POP exposures may hinder adaptive capacities and resilience of marine taxa and assemblages to the long and short-term consequences of climate change and its many indirect consequences, such as elevated storminess, sea level rise, and heat waves^31–33^. Thus, these types of combinatorial stressors may elicit unpredicted responses and emergent effects not previously seen but that continue to be poorly understood^31,32,34–36^.

To aide in further evaluation, this analysis presents a systematic review of the evidence of the combined consequences of differing climate change drivers on the marine environmental fate and biological effects of POPs listed to the Stockholm Convention (see Table 1). It informs efforts of the U.N. advisory body, Group of Experts on Scientific Aspects of Marine Environmental Protection, Working Group 45^37^. Much of the current evidence of the climate-POP nexus applicable to the marine environment comes from research in the Arctic, as well as from an emerging body of evidence from Antarctica. In addition to linkages of exposure to local and regional areas of usage, as globally circulating pollutants, the emissions and transport of POPs are dependent on atmospheric and oceanic conditions, as well as food web structures and other ecological drivers, all of which are being affected by climate change. Much of the research examining POP environmental fate and biological effects has focused on the influence of climate warming. Climate change increases in ocean acidification, altered salinity regimes, and deoxygenation (hypoxia) are also increasingly apparent and spatially varied^3,38,39^. However, combined responses of these climate factors with chemical exposures are much less well studied. Research continues to be weighted to examining environmental fate processes, with more recent focus on health effects extending in some cases to higher levels of biological organization. While observations of climate impacts on marine species and populations are widespread, effects are not uniform, and this will likely apply to chemical interactions as some taxa and populations may be differentially vulnerable or resilient to these interactions depending on age demographics, health status, location, and temporal sensitivities^7,25,28,29,40–42^. Because recent assessments have evaluated POP environmental fate processes impacted by climate change, this review generally highlights key findings and refers readers to these more recent efforts. For biological effect pathways, a more detailed accounting is presented as these responses have been subject to less focus for the climate-POP nexus.

## Scientometry and conceptual model

**Fig. 1 |:**
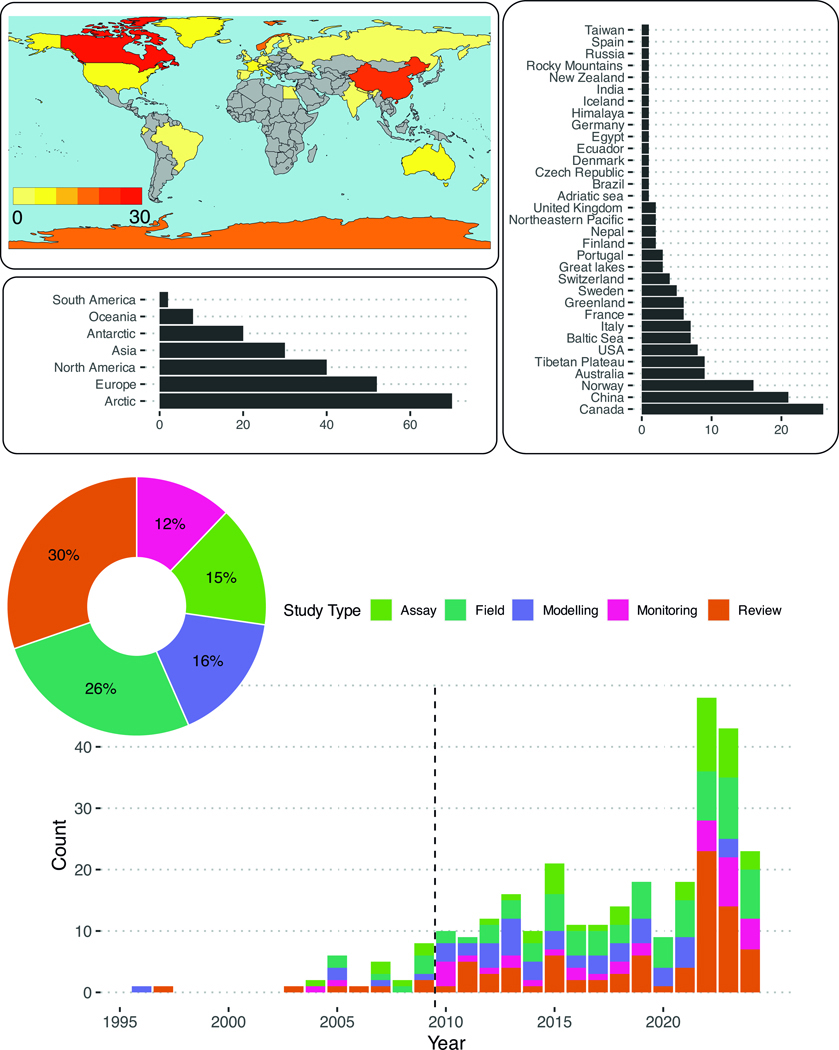
Scientometric analysis of the climate change-POP database by location, study type, and frequency. Dashed line denotes the generally limited research prior to 2010.

**Fig. 2 |:**
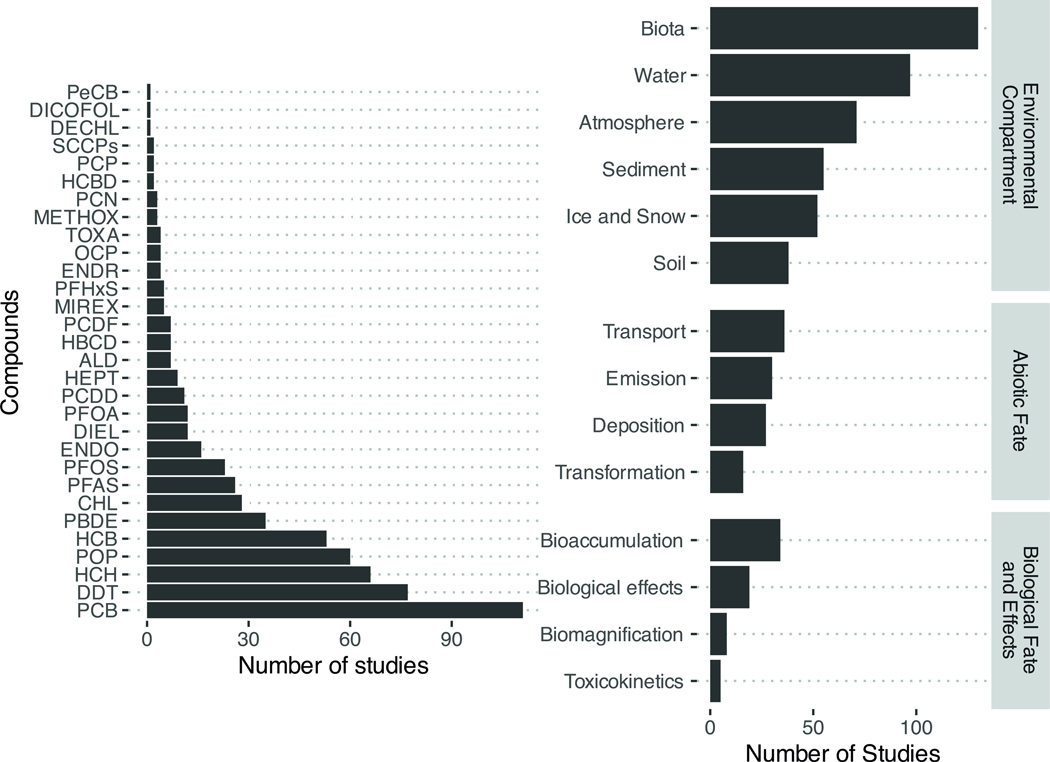
Scientometric analysis of the climate change-POP database by POP and topic. Studies may be tagged under multiple POPs and topics. ALD Aldrin, CHL Chlordanes, DDT Dichlorodiphenyltrichloroethane, DECHL Dechloranes, DIEL Dieldrin, ENDO Endosulfan, ENDR Endrin, HBCD Hexabromocyclododecane, HCB Hexachlorobenzene, HCBD Hexachlorobutadiene, HCH Hexachlorocyclohexanes, HEPT Heptachlor, METHOX Methoxychlor, OCP Organochlorine pesticide, PBDE Polybrominated diphenyl ethers, PCB Polychlorinated biphenyls, PCDD Polychlorinated dibenzo-*p*-dioxins, PCDF Polychlorinated dibenzofurans, PCN Polychlorinated naphthalenes, PCP Pentachlorophenol, PeCB Pentachlorobenzene, PFAS Per and polyfluoroalkyl substances, PFHxS Perfluorohexane sulfonate, PFOA Perfluorooctanoic acid, PFOS Perflourooctane sulfonate, SCCP Short chained chlorinated paraffins, POP Persistent organic pollutant, TOXA Toxaphene.

The 254 considered papers in this review were classified as laboratory assays (*n* = 46), monitoring (*n* = 37), modeling (*n* = 49), field studies (*n* = 79), and reviews (*n* = 89) (Fig. 1). There were 54 studies that examined patterns of shifting global distributions of POPs with climate change. Of regionally specific studies (*n* = 193), the majority (*n* = 167) targeted northern latitudes with much less evaluation in the Southern Hemisphere, mostly Antarctica. The selected studies also varied by environmental compartment with environmental fate studies largely focused on biota (*n* = 130), water (*n* = 97) and atmospheric (*n* = 71) processes (Fig. 2). Environmental fate studies covered global migration of POPs related to transport (*n* = 36), emissions (*n* = 30), deposition (*n* = 27), and transformation (*n* = 16). Studies on POP bioaccumulation (*n* = 34) and biological effects (*n* = 19) were also relatively well-represented. Most studies focused on the legacy POPs, notably the polychlorinated biphenyls (PCBs), *p,p*’-dichlorodiphenyltrichloroethane (DDT) and some of its metabolites (*p,p*’-dichlorodiphenyldichloroethylene (DDE), *p,p*’-dichlorodiphenyldichloroethane (DDD), hexachlorocyclohex anes (HCHs, including lindane), and hexachlorobenzene (HCB). There was little to no reporting on the more recently listed POPs (e.g., short-chain chlorinated paraffins (SCCPs), methoxychlor, dechlorane plus, UV-238).

**Fig. 3 |:**
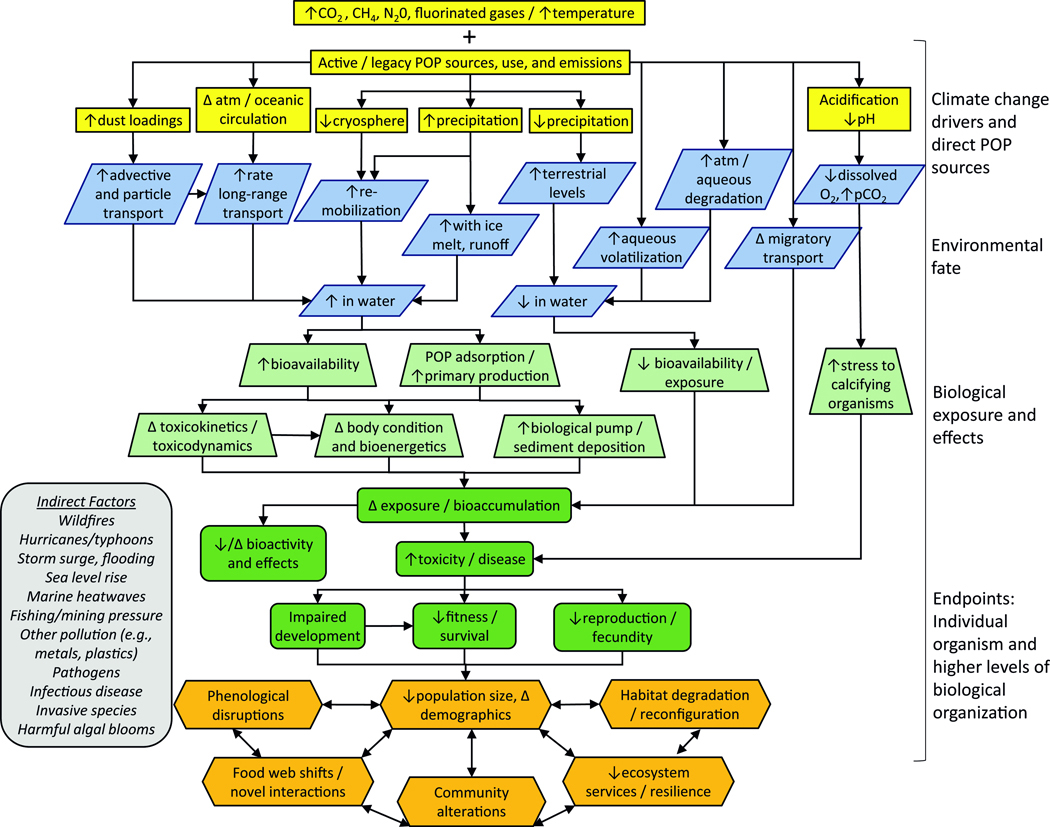
Conceptual model mapping database of combined climate change and POP stressors. Rectangular boxes (yellow): Climate change drivers and POP exposures as interacting primary (legacy contamination, ongoing use, climate warming) and secondary (cryosphere melting, glacial retreat, transport) sources and stressors. Parallelograms (blue): Environmental fate of POPs arising from interacting primary and secondary climate stressors. Trapezoids (light green) and rounded boxes (dark green): Resulting cascade of shifting exposure and biological effect pathways. Hexagons (orange): Continuing effects extending to higher levels of biological organization with two-way arrows depicting likely interacting feedback. Indirect factors (gray box): Abiotic and biotic modifying factors potentially influencing sources, stressors, and biological responses.

Based on the available evidence, a high-level conceptual model is presented in Fig. 3 mapping empirical and putative relationships of ecological receptors (e.g., invertebrates, fish, marine mammals) and attributes (e.g., reduced survival, altered food chains) to climate change shifts in POP exposures and effects. Climate change drivers (e.g., increasing temperature, altered precipitation, and atmospheric circulation [yellow boxes]) are influencing POP transport and fate processes in some regions (Blue parallelogram). This shifting biogeochemistry is prompting potential alterations in POP exposure and effect pathways [green trapezoid and rounded boxes]. These combinatorial stressors may affect biological receptor responses, such as increasing POP bioavailability and bioaccumulation, altering metabolic and bioenergetic responses, and increasing toxicity that in turn may impact individual organisms, populations, and communities (orange hexagon boxes). Downstream impacts moving to higher levels of biological organization are inherently difficult to predict with overlapping feedback responses and consequences that influence each other as depicted by the two-way arrows. It is also the case that sources, stressors, and biological responses in the conceptual model can be influenced by differing indirect modifying factors (Grey box/italicized text). This type of schematic is a high-level example that is useful for multi-stressor ecological risk assessment^43^. It does not depict all potential combinatorial responses, and as discussed below, the database is geographically, chemically, and biologically limited. Nonetheless, it can begin to array complex and variable sources and stressors to ecological endpoints of concern^44^. Thus, a road map begins to emerge in identifying pathways and endpoints by which ongoing chemical exposures interacting with climate change may produce adverse effects with an eye towards research and decision making.

## Environmental fate

Climate change is altering the biogeochemistry of the marine environment, that is in turn influences the environmental fate of POPs by direct and secondary (e.g., altered atmospheric and oceanic circulation) processes that are modified by indirect factors (e.g., extreme weather, sea level rise, wild-fires; see Fig. 3)^7,20,27,45,46^. Most research of POP interactions with climate change has occurred in the Arctic and Antarctic, where the magnitude of climate shifts is the greatest and occurring most rapidly^20,29^. There have been a number of large-scale evaluations of these interactions, including by the Arctic Assessment Monitoring Program (AMAP; see Table S.2) and several reviews^4,25,27,29,45,47–50^. Thus, as indicated earlier, topics in this section are discussed generally at an overview level with reference to more comprehensive reviews.

Cryosphere melting^51–60^, atmospheric transport and deposition^61–67^, and sediment remobilization^46,59,68–74^ are climate change processes mediating shifts in POP concentrations from existing Arctic reservoirs in abiotic and biotic compartments. As shown in the conceptual model (Fig. 3), several direct and secondary abiotic processes are reported that may enhance or reduce POPs levels over time^20,75^. For example, melting sea ice, snowpack, and glaciers containing POP reservoirs across differing timescales can accelerate delivery to the marine environment, whereas POP concentrations in surface waters can be reduced with volatilization and increasing air-seawater exchange with rising ocean temperatures^20,27,48^. Studies and modeling of climate and time series trends report remobilization of PFAS due to ice melt^76–78^. Increasing PFAS levels with cryosphere melting have also been observed in sediment cores of the Canadian Arctic^79^. However, for legacy chlorinated POPs, biomonitoring of planktonic populations in fjords of the Arctic Svalbard region (Norway) found increasing glacial melting to be associated with POP reductions, although small increases in α-HCH have been reported^80^. These differences across POPs suggest that glacial melting may be a more relevant secondary source for newer POPs, such as the PFAS, than for legacy POPs that are no longer in widespread use, although this remains to be studied in more depth.

In some agreement with glacial monitoring results, global-scale multimedia modeling suggests that climate change influences on the long-range global transport of legacy POPs to the Arctic is modest relative to changes in POP emissions over similar time horizons^20,27,81^. Important uncertainties remain, however, including that modeling continues to be mostly constrained to a subset of legacy POPs (PCBs, α-HCH, and *p,p*’-DDT). Another uncertainty is that this modeling tends not to capture greenhouse gas emission scenarios that may lead to non-linear feedbacks and climate tipping points (i.e., abrupt alterations in the magnitude of a climate variable, such as marine heatwaves). Indeed, impacts from indirect modifiers (see Fig. 3) are considered to have greater potential consequences than direct and secondary pathways, but are much less well characterized and predictable at present^20^. For example, the increasing frequency and severity of extreme weather events leading to flooding contributes to increasing runoff that mobilizes and deposits chemicals to the aqueous phase that may enhance bioavailability to marine food webs^45,82,83^. Another indirect pathway of new POP emissions relates to increasing wildfire activity with climate change, which increases atmospheric levels of PCBs and other POPs that may be deposited to aquatic systems^84^.

Understanding climate change impacts on the environmental fate of POPs is also continuing to evolve with the emergence of relatively newer chemical classes like the PFAS that exhibit differing physicochemical properties (e.g., comparatively higher water solubilities) and, like legacy POPs can undergo long-range atmospheric and oceanic transport^85^. However, there continue to be global disparities in monitoring and a focus on mostly legacy chlorinated POPs such as PCBs, while other chemicals are less represented both spatially and temporally^42^. This uneven distribution makes the analysis of trends difficult, particularly given the relatively large gaps in monitoring of Southern Hemisphere marine systems. It is also the case that some of the declining trends of some legacy POPs have leveled off or reversed in some environmental media, suggesting climate shifts may be playing a role in these changing fate dynamics^61^. For example, increasing atmospheric concentrations of some PCBs and *p,p*’*-*DDT have been observed in the Arctic, and are thought to be attributable to increasing volatilization with rising ocean temperature and snow and ice melt^20^. Other testing of Arctic (and Antarctic) marine sediments measured elevated levels of pentachlorophenol (PCP) and polychlorinated dibenzo-*p*-dioxins/furans (PCDD/F) comparable to sediments in more heavily industrialized mid-latitudes (South Baltic Sea) with the highest levels near retreating glaciers^86^. Additionally, increases in primary productivity in Arctic waters due to rising temperature are accelerating the transport of POPs from surface waters to deep waters and sediments (‘biological pump’ process)^45^. Rising ocean temperatures may increase mesoscale ocean eddies responsible for mixing heat, carbon, and nutrients in the water column, resulting in greater contaminant loads to deeper environments^87^.

While not as well studied as climate-POP interactions in the Arctic, POP occurrences in Antarctica appear primarily attributable to long-range atmospheric transport mechanisms that make this area a final deposition point^29,88–91^. However, evaluations report mostly lower levels of POPs in the Antarctic than in the Arctic, although long-term temporal trends data are scarce for Antarctica, and climate change factors are generally not well characterized^92^. Additionally, local emissions of some organochlorine POPs from human activities, such as emissions from the continuing use of Dicofol and technical DDT pesticides in South America and South Asia also contribute^93,94^. Ice melt as a source of some POPs (HCHs, DDTs) to Antarctic coastal lake waters has been investigated using sediment samples from King George Island^95^. Sedimentary levels of ∑DDTs were 28.51 ng g^−1^ dry weight (mean) in the melting glacier-fed lake, in comparison to 2.5 ng g^−1^ dry weight in the lake fed only by snow, rain, and penguin guano. Additionally, the release of stored POPs from melting ice into coastal waters over a 30-year period (1980–2011) has been reported to play a role in increasing concentrations of ∑DDTs and ∑polybrominated diphenyl ethers (PBDEs) in Ross Sea populations of Antarctic stenotherm fish *Trematomus bernechii*^96^. Similarly, evaluation of the air-seawater exchange of organochlorine pesticides, PCBs, and HCHs in Fildes Bay, Antarctica, indicates POP remobilization from the cryosphere with climate warming and glacial retreat that may promote these areas as secondary sources of more volatile POPs^97^. However, temperature-induced increases in POP remobilization to the atmosphere from Antarctic soils was observed to decline with an associated 0.5% increase in soil organic matter, illustrating that climate change factors may counteract each other^98^.

Studies in temperate and tropical zones are scarce, focusing mostly on shifting POP distributions from contaminated sediments with rising temperatures and extreme events (e.g., flooding)^99–101^. For example, increasing concentrations of ∑PCBs, ∑DDTs, and ∑HCBs in biopsy samples of humpback and snubfin dolphins from inshore Queensland, Australia populations were associated with increasing flooding and runoff from surrounding catchments^99^. A mesocosm experiment of POPs in a brackish water system has also shown that elevated dissolved organic carbon with runoff favors the sedimentary accumulation of *p,p*’-DDT^100^. Similar positive associations of sedimentary accumulation of POPs with DOC are also reported in the Baltic Sea^62^. However, other studies suggest less of a role for POP partitioning to dissolved organic carbon outside of coastal zones^102^. The link of POP fate to the carbon cycle in the ocean has been well stablished, and changes in the ocean carbon cycle will impact the behavior of POPs in processes such as sedimentation and resuspension^103^.

POPs emitted at temperate and tropical latitudes undergo global distillation along latitudinal gradients, with air-seawater exchange and deposition patterns that depend on their volatility and local temperatures. Furthermore, the increasing frequency and intensity of extreme weather events, such as summer monsoons and typhoons/hurricanes, are predicted to enhance atmospheric transport and runoff of POPs from emission area reservoirs^104^. The continuing use of technical DDT in tropical regions for malarial control and its volatilization from contaminated soils results in emissions and global transport to polar regions^93,94,105^. Modeling of the environmental transport of α-HCH in China from 1952–2009 reported outflows by the Northeast Boundary and the Mid-South Boundary, and suggested a potentially stronger outflow through the Northeast Boundary with the Eastern Asian summer monsoon^67^. Additional modeling of the Chaohu watershed (China) indicated climate increases in precipitation and atmospheric deposition, leading to increases in POPs in water and soil^54^. High levels of some PFAS, PCBs, and other POPs were also found around coastal areas of the Bohai Sea and Yellow Sea^106^. Bohai Rim is a highly urbanized and industrialized area in the Bohai Sea, China. Modeling atmospheric deposition of perfluorooctane sulfonate (PFOS) for that area predicted that climate change would decrease concentrations in freshwater and urban soils whereas increasing trends would occur in coastal waters and rural soils^107^.

A global simulation of the fate of PCB congeners noted that rising temperatures, changing circulation, and sea-ice loss modeled over 1992–2015 has led to greater losses of the lighter PCB-28 congener from the Arctic but increased net deposition and stability of the relatively heavier PCB-153 congener likely due to particle sorption^102^. Moreover, this modeling also showed Northern Hemispheric oceans to be net exporters of historical contaminants to the Equatorial and Southern Ocean basins for several decades. Another example of this directional transport is provided by field sampling of different global regions (e.g., Atlantic Ocean, Greenland Sea, Southern Ocean) that shows the Arctic to be a source of PFAS, possibly due to remobilization in melting snow and sea ice^108^.

## Bioaccumulation

Tables 2 and 3 summarize time series studies evaluating the influence of differing climate and ecological parameters on POP temporal trends in Arctic/subarctic and Antarctic biota, respectively. While the tables are not intended to be an exhaustive accounting of all studies, the results demonstrate a generally mixed picture concerning the influence of climate change on POP bioaccumulation trends. Overall use reductions appear to be decreasing bioaccumulation of legacy POPs in the Arctic, with climate change influencing these trends depending on location and species^20,25,27,49,109^. While biomonitoring efforts in the Southern Hemisphere are scarce, a small number of studies have evaluated climate-related impacts on bioaccumulation trends in Antarctic organisms with similar mixed results^20,27,29^.

Current climate evidence supports generally positive Arctic Oscillation (AO) and North Atlantic Oscillation (NAO) trends being partly influenced by increases in greenhouse gases and natural forcings^110–113^. Positive correlations of the winter AO and NAO index to increasing POP bioaccumulation trends in Arctic species are reported, possibly due to influxes of atmospheric and oceanic currents from North America and Europe, although this remains to be further characterized (see Table 2)^20,114^. Additional ecological factors also appear to be operating at more restricted spatial scales, producing distinct temporal characteristics (e.g., winter foraging, spring breeding; see section on Phenological and Food Web Shifts). Other factors, such as the timing of sampling are important, particularly among breeding female seabirds and marine mammals that may transfer POPs to eggs and offspring, respectively^25,115–118^. However, the database continues to be limited and heterogenous in terms of temporal and species coverage, with large geographical gaps, including a lack of analysis of mid and tropical latitude ecosystems.

Altogether, efforts focused principally on Arctic environments have contributed to describing observed and projected alterations in POP global cycling with climate change. These efforts also inform how these processes may operate as climate change advances in Antarctica and more temperate and tropical regions, where POP use and emissions may be higher. However, it continues to be challenging to forecast climate change influences on POP bioaccumulation due to the spatial and species variability and ongoing uncertainties of modeling.

## Toxicokinetics and bioactivity

Numerous health effects have been shown with POP exposures, including cancers and disorders of the nervous, reproductive, endocrine, liver, and immune systems^6,10,22,119–122^. However, only a limited number of studies have examined the consequences of differing climate drivers on the toxicokinetics and bioactivity of POPs to marine biota, mostly restricted to survival in fish (see Table 4). Toxicokinetics by processes of absorption, distribution, metabolism, and excretion informs how an external concentration (e.g., dietary) of a chemical will translate to internal concentrations that can lead to toxicity. It generally depends on interacting factors of chemical physicochemical properties, species, and environment.

While increasing temperature is hypothesized to generally increase the bioavailability and toxicity of contaminants, the database continues to present an unclear picture with potentially competing toxicokinetic processes that may involve increased uptake and metabolic activation counteracted by elevated detoxification and clearance^5,31,33,123–128^. For example, an increase in the bioaccumulation of PCBs and *p,p*’-DDT in the Arctic fish burbot (*Lota lota*) was shown over a 21-year period from 1988 to 2008, and was unexpected due to decreasing atmospheric levels of PCBs with use restrictions^125^. The study postulated that elevated primary productivity with climate change at higher latitudes potentially increased POP adsorption to organic matter and enhanced bioavailability. While not examined in this study, increases in bioaccumulation may then influence patterns of metabolism and excretion that have also been shown to be affected by temperature. For example, toxicokinetic testing of PCBs in rainbow trout (*Oncorhynchus mykiss*) has shown increased production of more bioactive and persistent hydroxy PCB metabolites with rising ambient temperature but also decreasing biological half-lives of other PCB congeners^129^. Consistent with some of these findings, a toxicokinetic study in northern leopard frog tadpoles (*Lithobates pipiens*) found that rearing temperature had no significant effect on tissue levels of two PCB congeners (PCB-70, PCB-126) or the pentaPBDE commercial mixture DE-71 due to estimates of both faster uptake and elimination of the tested POPs with increasing temperature^123^. Toxicodynamics were not evaluated in these studies in fish and amphibians.

In terms of bioactivity, elevated ambient temperatures increased the toxicity of endosulfan (1.5 μg L^−1^) in the warmwater fish, silver perch (*Bidyanus bidyanus*), with statistically significant increases in mortality (reduced half maximal effects concentration; EC50) after a 24-h exposure^130^. Reduced time to effects (i.e., time to 50% of effects; ET50) was also reported in silver perch and in coldwater rainbow trout co-exposed to endosulfan at elevated temperatures^130,131^. Toxicity screening assays in zebrafish (*Danio rerio*) embryos, which are models of vertebrate development, also observed adverse effects with co-exposures to elevated temperature (35 °C) and endosulfan (50 μg L^−1^)^132^. Embryos exposed to endosulfan under elevated temperatures exhibited incomplete or impaired brain morphology, regionally specific inhibition of brain gene expression, and enhanced stress gene responses, whereas null effects were reported with exposures to increased temperature or endosulfan alone.

Rising temperatures also have been shown to affect POP tissue distributions. For example, rising ambient temperatures resulted in an increase in the distribution of PFOS and perfluorohexane sulfonate (PFHxS) in blood, liver, and brain (and decreased concentrations in muscle) of rainbow trout receiving a 42-day dietary exposure^133^. This study observed rising temperatures enhancing the clearance half-lives of both PFAS, with mixed results across other organs that varied by temperature and compound, but nonetheless demonstrated the importance of temperature-related distribution mechanisms influencing body burdens^134,135^. There are also data suggesting reduced effects with rising temperature, including reductions in the toxicity of *p,p*’*-*DDT to midges (*Chironomus dilutes*) at elevated temperatures that were postulated to be attributable to increasing neurological effects at lower temperatures^136^. However, exposing the zooplankton *Daphnia magna* simultaneously to a long-chain PFAS mixture at 60 and 120 μg L^−1^ and elevated temperatures (16, 24 °C) in a standard 48-h bioassay resulted in synergistic effects on immobilization in comparison to either the PFAS mixture or temperature exposures alone^137^.

To facilitate toxicokinetic evaluations, an increasing number of modeling tools hold promise for higher tiered assessments of multi-stressor dynamics, including by integrating temperature dependencies using calculated changes in reaction rates (e.g., Q10 temperature coefficients, Arrhenius and Van’t Hoff equations)^123,126,138–142^. In the context of POPs, a dynamic energy budget model with empirical testing was used to evaluate the toxicokinetics of PCB-153 and PFOS in juvenile common sole (*Solea solea*) residing in breeding grounds of the Gironde estuary (France)^141^. This study found dietary composition to have a statistically significant effect on fish growth and body burdens of the POPs tested, whereas temperature affected growth alone. There have also been efforts to develop modeling approaches to evaluate the mass transfer of some POPs to pelagic marine mammals^143,144^. A nutrient-phytoplankton-zooplankton-detritus model was developed, accounting for temperature-induced changes in plankton dynamics and resulting partitioning of HCB into krill^143^. This partitioning model was then integrated with a dynamic energy budget model of energy distribution and storage in the humpback whale along with a physiologically-based pharmacokinetic model of HCB body burdens^144^. While not specifically linked to climate change at this time, it demonstrates possible avenues for advancing empirical testing and toxicokinetic modeling strategies.

Taken together, the influence of climate warming on POP toxicokinetics and effects is chemical and organism-specific. Although the limited database tended toward increasing uptake and bioactivity of some POPs with temperature, determining overall trends was not possible given the limited scope of the database.

## Thermal tolerances and bioenergetics

Responses to increasing temperature and chemical exposures are influenced by the extent to which a population is residing at or near its temperature tolerance limits. Ectotherms are adapted to relatively narrow windows of ambient temperature, whereby excess warming can exceed the aerobic capacity of mitochondria and proceed to less efficient and sustainable anaerobic energy production, in addition to other effects^145^. Thermal safety margins and thermal performance curves are strategies used to evaluate the effects of thermal changes on physiological responses and adaptation traits^146–150^. They have also been applied to a lesser extent in examining shifting thermal tolerances in the context of temperature-chemical exposures^145,150,151^. Thermal safety margins describe a thermal tolerance ratio of the maximum temperature tolerable (critical thermal maximum; CTmax) to actual environmental ranges. Thermal performance curves evaluate temperature effects on species performance, conventionally applying physiological (e.g., growth, oxygen consumption, cardiac functioning) or fitness (e.g., survival, reproductive output) responses.

Short-term 96-h testing in marine medaka (*Oryzias melastigma*) larvae showed elevated seawater temperatures in combination with increasing *p,p*’*-*DDT exposure to reduce thermal tolerance ranges relative to seawater-solvent controls containing no *p,p*’*-*DDT^152^. Thus, a *p,p*’*-*DDT-induced temperature sensitivity emerges whereby some marine fish exposed to *p,p*’*-*DDT appear to have less short-term capacity to acclimate to thermal variations than populations exposed to thermal change alone. Similarly, testing with endosulfan showed reductions in thermal tolerances (i.e., decreased CTmax) and an increase in toxicity with rising temperatures particularly in cold water rainbow trout^130,131^. Other testing reported that an 18-h exposure to endosulfan and increasing ambient temperatures did not have a statistically significant effect on gill ventilation frequency of silver perch, although the short exposure duration complicates interpretation^153^. Comparative analyses of differing thermal regimes have also suggested that tropical marine ectotherms may be more susceptible to climate warming given their elevated baseline body temperature and narrower thermal safety margins^154–157^. However, the role of POP exposures in affecting responses of tropical marine biota to climate warming remains unstudied.

Another example of adverse responses is provided by the elevated oxidative stress observed in some thermally stressed corals exposed to POPs. The Scleractinian coral *Stylophora pistillata*, native to the Indo-Pacific and a common research model, was exposed to PFOS for 28 days under standard and elevated temperatures^158^. In the absence of elevated temperature, PFOS exposures alone did not significantly affect survival, photosynthesis rates, *Symbiodinium* densities, or pigment content (*Chlorophyl A*). However, at elevated temperatures, there were decreases in symbiont densities and photoprotection mechanisms. Adding PFOS exposures under elevated temperature prompted additional physiological impairments including reduced photosynthetic efficiency and net photosynthesis rates, as well as increased generation of oxidative stress biomarkers. This response suggests exposure to PFOS may potentiate the effects of climate warming oncorals by exacerbating bleaching (i.e., expulsion/digestion of endosymbiont) with photoinactivation and/or oxidative stress^159^.

In addition to examining thermal adaptation responses of marine species, there has been some evaluation of the effects of climate warming and POP exposures on sublethal stress and bioenergetic responses, including in habitats with differing pollutant loads. Specifically, the effects of elevated temperature were evaluated in juvenile European flounder (*Platichthys flesus*) collected from the Seine Estuary (France) and Vilaine Estuary (France), representing highly polluted and moderately polluted ecosystems, respectively (e.g., ∑PCBs and ∑PBDEs about 10X higher in the Seine than Vilaine)^160^. This study showed that flounder collected from the more polluted Seine and acclimated to elevated temperatures were significantly reduced in size with declining expression of genes involved in mitochondrial energy production in comparison to fish from the Vilaine subjected to identical thermal regimes. These findings were replicated and added to in a study that also showed this pattern of bioenergetic deficits in juvenile European flounder from the heavily polluted Seine, as well as in populations residing at its southern thermal limit (Mondego Bay, Portugal)^161^.

Related testing in other species indicates altered energy budgets and reduced fat content in juvenile seabream (*Diplodas sargus*) exposed by diet to elevated temperatures and the PBDE flame retardant decabromodiphenyl ether (BDE-209)^162^. Additional behavioral studies in juvenile sea bream also reported that dietary exposure to BDE-209 in the presence of elevated temperatures and/or acidification resulted in extended risk aversion responses consistent with increased anxiety, whereas exposures to BDE-209 alone resulted in opposing hyperactivity behaviors interpreted as reduced risk awareness^163^. While the environmental conditions studied (acidification and/or warming) may have dominated the combinatorial responses, results suggest that combinations of warming, acidification, and BDE-209 exposures may prompt maladaptive behaviors that influence predation, foraging, and socialization.

Studies in Arctic and subarctic species reported POP-induced disruptions in thyroid hormones involved in bioenergetics, thermoregulation, and development, as well as other endocrine system perturbations potentially influenced by climate change^8^. Increasing temporal trends of PFAS have been measured in eggs and livers of thick-billed murres (*Uria lomvia*) colonies in the Canadian Arctic^164,165^. Serum levels of long chain PFAS were associated with reductions in the body mass of Hudson Bay (Canada) murres^166^. This study also reported perturbed thyroid hormone homeostasis and reproductive fitness (hatching success, incubation efficiency), potentially influenced by effects of climate warming on bird diets, body condition, and stress responses. No associations were reported for other POPs examined (∑PCBs, *p,p*’-DDE, ∑PBDEs, HCB)^167^. However, the authors note that the timing of sampling after the breeding season suggested maternal transfer of POPs to eggs that may have biased results. Studies also show impacts of increasing ice melt on the bioenergetics of some Arctic marine mammals (polar bears), resulting in the release of lipid sequestered POPs into circulation with fasting and altered metabolic demands, leading to potential reduced fitness and reproductive capacity^25,49,168^. Altogether, the evidence supports that some marine mammals, seabirds, and ectotherms residing in POP-contaminated environments and/or in combined temperature edge habitats may experience declines in body condition, fitness, energy storage capacity, and reproductive performance with climate warming.

## Effects of acidification and salinity change

Increasing ocean carbon dioxide and acidification (quantified as the sea surface partial pressure of CO_2_; pCO_2_) are notable climate stressors and particularly deleterious to calcifying marine organisms, suchas Scleractinian corals, echinoidea, crustacea, and mollusks that synthesize calcium carbonate to produce shells and other skeletal structures. However, just one study with POP exposures in corals has evaluated the effects of PFOS in the presence of elevated temperature^158^. There has been very little examination of more environmentally relevant scenarios of acidification in the presence of climate warming with POP exposures. An exception is a study that exposed Mediterranean mussel (*Mytilus galloprovincialis*) and Manila clam (*Ruditapes philippinarum*) to the dechlorane flame retardants (Dec-602, −603, −604), perfluorooctanoic acid (PFOA), and PFOS with increasing temperature and decreasing pH, and in combination^169^. After a 20-day exposure, acidification alone decreased bioaccumulation of PFOA and PFOS in mussels. Notably, however, the bioaccumulative decline with pH reductions was eliminated for PFOS (not PFOA) when combined with increasing temperature. In contrast, generally increasing bioaccumulation of dechloranes was observed in clams in the presence of acidification or elevated temperature alone and when combined. Another study in breeding sea urchin (*Paracentrotus lividus*) co-exposed to acidification (pH 8.1, 7.7) and PFOS (0.5 μg/L) for 5 and 30 min reported decreased sperm motility and fertilization success with acidification alone, and no contribution of PFOS, although inferring effects from the short exposure duration is difficult^170^.

Regarding non-calcifying organisms, effects of increasing acidification are poorly understood and suggested to be mediated by elevated blood pCO_2_ (hypercapnia) rather than associated declines in pH^171^, with similarities in physiological responses to hypercapnia and low tissue oxygen (hypoxemia)^172^. In addition to widespread mortality events that can occur with marine environmental hypoxia and anoxia, it has been shown that low oxygen alone may impair reproduction and development by disrupting the endocrine system absent chemical exposures^31,172^. A short-term study in Atlantic cod (*Gadus morhua*) exposed to PFOS for five days, followed by a 9-day exposure to hypercapnia reported that hypercapnia alone, with lesser contributions when combined with PFOS, increased muscle tissue levels of 17β-estradiol, testosterone, and 11-ketotestosterone^173^. While this study did not evaluate internal hypoxemia, results differed from other testing in hypoxia-exposed fish showing decreased sex steroid hormones and other reproductive biomarkers (e.g., fecundity, growth, vitellogenin egg shell protein)^31^. The previously discussed 56-day study in juvenile sea bream exposed to BDE-209 (see section on Thermal tolerances and bioenergetics) reported that co-exposing fish to acidification alone (pCO_2_ = 1500 μatm) and in the presence of elevated temperatures elicited behaviors consistent with elevated anxiety^163^.

The IPCC also notes that large-scale, near-surface salinity contrasts are intensifying with climate change, the Pacific and Southern Oceans are freshening, and the Atlantic Ocean and coastal zones (e.g., tidal flats, marshes, and estuaries) are generally becoming saltier with sea level rise^1,174^. The only testing of combined effects of altered salinity in the presence of POPs was a 12-day study in adult tilapia (*Sarotherodon melanotheron*)^175^. This study evaluated the combined effects of waterborne exposure to *p,p*’*-*DDT under freshwater, seawater, and hypersalinity conditions. Short-term *p,p*’*-*DDT exposures inhibited gill NA^+^/K^+^-ATPase (NKA) activity and cystic fibrosis transmembrane conductance regulator chloride channels in saltwater-adapted fish compared to freshwater controls. Thus, it is possible that chronic exposures to low levels of *p,p*’*-*DDT in the presence of more frequent and severe salinity fluctuations may lead to osmoregulatory impairments. However, there has been little to no study of these interactions in other species. Nor has there been a study under longer exposure conditions, or in cartilaginous fishes that employ differing osmoregulation strategies.

## Ecological resilience and adaptation

Characterizing ecological resilience and adaptation when moving from individual organisms to larger, more complex ecological scales is challenging but integral to identifying important modifying factors, species, and environments vulnerable to the climate-POP nexus (see Fig. 3)^5,7,25,40,44,176^. The effects of climate change alone in reducing the resilience of marine populations and ecosystems are now well documented^3,177–180^. Time series studies of biota from the Arctic and Antarctic have been evaluating climate and ecological influences on bioaccumulation trends (e.g., see Tables 2 and 3). However, though progress is apparent, it remains difficult to assess combined chemical and nonchemical stressor interactions in propagating effects that may lead to adversity beyond the individual organism^34,41,128,181–183^.

Factors influencing ecological resilience and adaptation to the combined impacts of climate change and POPs are driven by the properties of the chemical, climate parameter, and season of occurrence in combination with other indirect modifying factors, as well as organism life history, distance to recolonization, and dispersal traits (see Fig. 3)^32^. An analysis of approximately 170 studies targeting marine ecosystems where two or more climate and/or chemical exposures were examined found responses in populations weighted to synergistic effects (effects greater than aggregate of individual components) that worsened as stressors accumulated^34^. Studies and reviews also posit that higher food chain predators, migratory species, and populations in edge, fragmented, and/or reconfigured habitats, or subject to other stressors may have heightened vulnerability^5,31,32,176,184–189^. Indirect responses to climate drivers (see Fig. 3), such as worsening storm events and wildfire activity may lead to higher pollutant loads that increase exposure and adverse effects, and depending on frequency and intensity, prolong or fail to allow adequate recovery time^32,190^.

However, these processes are not straightforward, and for example, it is possible that density-dependent processes such as reduced competition for resources could lessen impacts to some populations and communities than observed empirically among individuals^176,191^. It is also possible that indirect modifying factors could play a disproportionate role relative to the immediate climate and POP stressor in modifying overall health. Thus, for instance, populations and community assemblages subject to elevated POP exposures in warming environments may be more susceptible to pathogens and disease vectors that are themselves undergoing range shifts with climate change^189,192–197^.

As with chemical exposures alone, the duration of chemical and climate stressor modification are relevant factors in ecological resilience and adaptation. Hazard profiles will vary depending on whether exposures occur chronically over lifetimes or multiple generations as opposed to shorter pulsatile and extreme events (e.g., elevating temperature trends translating to lifetimes, wildfire exposure over weeks/months, storm events/runoff over days)^198^. Additionally, the potential for delayed effects may occur in that climate alterations from an earlier year may mediate effects later as is shown with the correlation of increasing winter NAOs and AOs on future increases in POP bioaccumulation in some northern species (see Table 2)^180^.

The age demographics of a population are another important consideration due in part to the elevated susceptibility of younger animals as they are undergoing rapid growth and have not yet developed a full complementary system of processes to facilitate adaptation^199–202^. For example, struggles with re-establishing an Arctic char population in Lake Bourget (France) have been hypothesized to be attributable to climate warming in the presence of legacy PCB contamination. To explore this hypothesis, adult female char were exposed intraperitoneally to PCBs at concentrations similar to Lake Bourget (500 ng g^−1^, 1000 ng g^−1^) 30 days prior to spawning, with eggs and hatchlings exposed to elevated temperatures based on climate warming scenarios^202^. Synergistic effects of PCBs and elevated temperatures on age demographic, physiological, and behavioral responses were observed that depended on larval life stage (see Supplementary Table 4), providing a plausible explanation for the persistent deficits in the char population.

In striped marsh frog tadpoles (*Limnodynastes peronii*), reduced body length and increased predation were reported with increased egg-rearing temperatures and a 96-h endosulfan exposure, and were posited to be attributable to altered muscle growth^199^. Similarly, elevated temperatures with a dietary co-exposure that included the DDT metabolite *p,p*’-DDE and a pesticide mixture altered the expression of genes involved in lipid homeostasis, and at higher temperatures reduced the burst speed of juvenile chinook salmon (*Oncorhynchus tshawytscha*)^200^. Additional testing in temperature-stressed juvenile chinook salmon co-exposed to a *p,p*’*-*DDE/pesticide mixture also observed neuroendocrine and olfactory effects that impeded predator avoidance behaviors^203^. Altogether, these studies suggest that legacy POP exposures with climate warming may alter the maturation of developing animals, leading to reduced survival and other effects that may skew age demographics.

## Phenological and food webs shifts

While data are scarce, phenological mismatches associated with seasonal and interannual variation in climate change migrations, foraging and predator-prey relationships, and life history traits may affect population and community responses that influence POP exposures, bioaccumulation, and effects (Tables 2 and 3)^20,25,27,204,205^. Reduced population fitness with individuals in poorer condition is plausible if species are unable to adapt to an earlier onset of spring, migrations, and associated food and resource availability with climate change^25,36,206^. For example, the winter NAO index was used as a proxy for large-scale climate change over the period 1999–2019 to evaluate POP bioaccumulation patterns in eggs with early migrations of goldeneye duck (*Bucephala clangula*) in milder winters^207^. A positive association of the winter NAO with PFOA levels was observed with early migrations from more polluted coastal feeding areas in Norway to less polluted freshwater breeding grounds. However, this trend was not reported for the other POPs evaluated suggesting this climate factor was generally not an important driver of POP bioaccumulation in eggs in this study.

POP alterations of primary productivity at the base of trophic food webs also have been reported, including for example, a mesocosm study of a coastal northern ecosystem that found increasing temperature and HBCD co-exposures to alter the structure of the zooplankton community^208^. Another mesocosm study of coastal runoff reported general declines in bacterial abundance and diversity with exposures to increasing terrestrial dissolved organic matter and a POP mixture^209^. In vitro testing with the Arctic cyanobacteria, *Pseudanabaena biceps*, reported that combinations of short-term exposure to elevated salinity and temperature with the PBDE contaminant tetraBDE-47 reduced cell density^210^. Additional studies of glacial ecosystems show deposited POPs having selective pressures on glacial microbial communities, potentially influencing downstream deposition and effects with accelerated melting^211^. A small number of studies also indicate the influence of climate parameters on Arctic zooplankton communities including by altering energy transfer and favoring northward shifts of subarctic species that alter and/or increase POP bioaccumulation^205,212–214^. However, the role of climate change is difficult to interpret due to putative competing processes of increasing POP adsorption to algae and zooplankton with dilution in a larger pool of biomass^25,215^.

Additional evidence of shifting food webs comes from population studies of cold-adapted, high latitude marine mammals and seabirds generally feeding athigher levels of food webs^20,49,50,115,168,216–225^. Declines in sea ice habitat with climate warming appear to be altering POP exposure pathways, food/prey availability, and spatiotemporal foraging behaviors that lead to food web shifts. While the temporal trend data are variable (see Tables 2 and 3), increasing body burdens of POPs in cold-adapted species with high lipid reserves is likely partially attributable to reductions in bodyweight with fasting or with animals in poorer condition with climate change, which re-release previously lipid-sequestered POPs into the serum compartment. For example, increasing temporal trends of POPs (HCB, DDT, PCBs) in stranded male humpback whales migrating in the Southern Ocean were reported in years linked to reductions in sea ice and poorer feeding conditions^206,221^.

Because legacy POPs tend to biomagnify up food chains, animals feeding at higher trophic levels will be expected to accumulate POPs at higher levels. Trophic shifts and feeding patterns with climate change appear to be altering contaminant profiles in polar bear populations, including from the western Hudson Bay^219^, eastern Greenland^220,224^, southern Beaufort Sea^192,216^, and Svalbard, Norway^225,226^. For example, the timing of seasonal sea ice break-up in the western Hudson Bay from 1991–2007 was shown to be associated with higher POP concentrations linked to changes in seal prey species^219^. Some of the available studies of these responses in polar bears also show associated fitness deficits that include general declines in body size and condition that may in turn, impact other biological responses, such as immune signaling^192,216^ and energy metabolism^225,226^.

Climate warming shifts in dietary patterns among some Arctic seal populations are increasing accumulation of newer POPs (e.g., PFAS) but less so legacy organochlorine POPs, aligning more closely to POP bioaccumulation patterns in subarctic seal species and populations^227^. Declining POP levels with decreasing sea ice extent also have been reported in populations of ringed seals (*Pusa hispida*) from eastern and western Greenland^222^. Additionally, research of ringed seal populations along the Labrador coast (Canada) observed that a population occupying a narrow estuarine habitat had greater access to food resources but was potentially more vulnerable to sea ice loss and PCB exposures than a nearby population residing in a wide range of habitats of localized inlets to far offshore^204^. Another example is provided by Antarctic fur seal (*Arctocephalus gazella*) populations that are in steep decline due to a combination of anthropogenic stressors including POPs, other pollution, and climate-accelerated sea ice melt and glacial retreat^228^. In some alignment with these findings, New Zealand fur seals (long-nosed fur seal; *Arctocephalus forsteri)* populations monitored between 1998 to 2019 exhibited an increasing temporal trend of liver concentrations of some POPs (*p,p*’-DDE, PCB-153) associated with animals in poorer and emaciated body condition^229^. These results suggest that POP mobilizations with bioenergetic imbalances linked to food web shifts and/or impaired feeding may be influencing seal population ecology and, in some populations, increasing POP exposure and effects.

The associations of POP levels in seabird populations to ambient temperature show complex multi-stressor relationships particularly with food resources (Tables 2 and 3). For example, shifts in dietary patterns with climate change have been reported to reduce POP bioaccumulation trends in colonies of thick-billed murre (*Uria lomvia*) in the Canadian Arctic^115^. Research in reproducing glaucus gulls (*Larus hyperboreus*)^230^ and great skuas (*Stercorarius skua*)^231^ indicates POP levels in nutritionally stressed females to be negatively correlated with chick growth rates and survival, with the amelioration of effects in first born skuas with food supplementation. Testing of fasting common eider (*Somateria mollissima*) females breeding in the high Arctic (colder) showed birds to have reduced body conditions, with high lipid metabolism and elevated POPs in comparison to subarctic populations residing at higher temperatures^232^. Additional research also showed associations of air temperature with blood levels of *p,p*’-DDE, HCB, and PCB-153 in high arctic common eiders^233^.

Taken together, the weight of evidence supports that body burdens of some POPs in Arctic and subarctic species (and possibly Antarctic species, though there is less data) are being altered by climate shifts in dietary patterns, food webs, and hunting/foraging behaviors (Tables 2 and 3). However, interactions of climate-POP variables in marine species residing in colder environments are not consistent and demonstrate the complexity of both direct and indirect interactions of population responses to climate change that are expected to vary by species, life stage, population, and ecosystem.

## Migrating populations as POP vectors

With shifting migration patterns under climate change, there has been some attention on whether and how altering population movements may be acting as bio-vectors in the transport of POPs from contaminated to uncontaminated regions^5,7,25,184,234–238^. Most of the research in this area has been with seabirds. For example, sediment levels of HCB and *p,p*’-DDT were 10 to 60 times higher in Canadian coastal ponds under the nesting cliffs of northern fulmars (*Fulmarus glacialis*; via excretion) than in sediments from nearby ponds absent nesting birds and exposed by atmospheric deposition alone^234^. A study of Kentish plovers (*Charadrius alexandrines*) found levels of *p,p*’-DDT, HCH, PCBs, PBDEs, and HCB to be higher in birds immigrating to breeding grounds relative to the emigration from breeding grounds^237^. Norwegian Arctic lakes have been shown to have elevated levels of PCBs from the guano of migrating sea birds^235^. Likewise, elevated *p,p*’-DDT and HCH concentrations have been measured in Antarctic sediments at locations where Adélie penguins (*Pygoscelis adeliae*) historically migrated^236^. More recently, testing of POP levels in the tissue and guano of Adélie penguins residing in Antarctica relative to migrating polar skua (*Catharacta maccormicki*) showed PCB transport to Antarctica by skua, whereas newer POPs (PFAS, PBDEs, HBCD) were detected in both skua and penguins, suggesting exposure by other routes^238^. The sediments of subarctic lakes receiving sockeye salmon (*Oncorhynchus nerkus*) returns have also been shown to have PCB levels several fold higher than in nearby lakes subject to atmospheric PCB inputs only and comparable to areas with industrial releases^239^.

## Conclusions

The systematic review herein provides evidence that climate change is affecting the fate of POPs, and that contaminant exposure interactions with differing climate change drivers are influencing some wildlife health metrics. However, the database is insufficient to offer a definitive global assessment of current trends and biological responses. Progress has been made in understanding climate impacts on the environmental behavior of POPs in high-latitude Arctic and subarctic environments, but data gaps continue in southern polar, temperate, and tropical ecosystems. Data gaps also persist in understanding the biological and ecological effects of the climate-POP nexus on marine biota and assemblages. Here too, progress is apparent, but with most research focused on a narrow set of endpoints, species, and environments. Filling data gaps by exclusively testing and monitoring the extensive range of marine environments, species, and climate-chemical interactions will be impractical. More geographically extensive monitoring and predictive distribution models, such as Globo-POP^240^ may facilitate understanding potential future spatial trends in environmental distributions and regional sources of POPs with climate change. Furtherance of predictive tools and models (e.g., multi-criteria decision analysis, problem structuring methods, meta-analyses, adverse outcome pathways, Bayesian networks) to pair with testing strategies will also be important in identifying the species, populations, and communities most at risk. Such advancements will aide in evaluation and decision-making and hold the potential for substantial future societal and ecological benefits by protecting ecosystem services and functioning.

## Methods

**Fig. 4 |:**
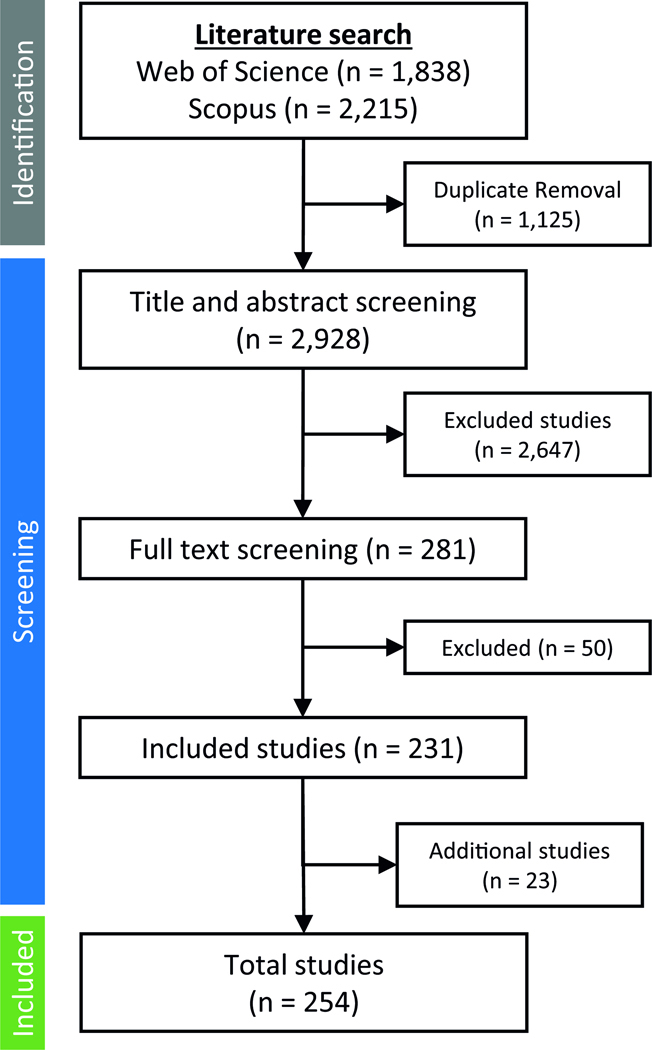
Literature search flow diagram for systematic review through August 2024.

The search strategy examined the scientific literature using two databases, Web of Science and Scopus, and is reported in accordance with the Preferred Reporting Items for Systematic reviews and Meta-Analyses^241^. See Supplementary Table 1 for Boolean strings and keyword search terms. Selected keywords were searched in the title, abstract, and keyword fields of both databases (no date limit to August 2024). Figure 4 presents the workflow of the literature search, and Table 1 identifies the POPs targeted in the search. Search terms included overarching POP and climate change terminology (e.g., organochlorine and greenhouse gas), as well as differing chemical nomenclatures and breakdown products. Several governmental and non-governmental reports have evaluated these topics to a varying extent, mostly in the Arctic. These are included in the review (See Supplementary Table 2 for a list and links to reports).

There were 4053 studies identified in the literature search that were downloaded for analysis in Excel. After duplicate removal, 2928 titles and abstracts were screened for inclusion/exclusion according to the following criteria: peer-reviewed article in English; title and abstracts mentioned the transport, fate, and biological effects of combined climate change drivers and POP exposures. Biological effects could include marine organism bioavailability, bioaccumulation, biomagnification, toxicokinetics, physiological effects, toxicity, adverse effects, mortality, bioenergetics, thermal stress, and population, community, or ecosystem-level responses. Based on these criteria, 281 papers were advanced for full text evaluation, after which an additional 50 papers were removed for not fitting the selection criteria. An additional 23 studies were identified from reviews and governmental reports, resulting in a total of 254 studies. Studies were screened and assigned tags for organization and review purposes based on geographic location, POPs evaluated, environment compartment and process, and biological effects. Tags were used for a scientometric analysis of the literature and to guide the review.

## Supplementary Material

Supplement

**Supplementary information** The online version contains supplementary material available at https://doi.org/10.1038/s43247-025-02348-4.

## Figures and Tables

**Table 1 |:** Stockholm Convention Persistent Organic Pollutants (POPs) included in literature search^a^

Chemical	Primary sources			Annex^b^
	Pesticide	Industrial, consumer	Unintended byproduct	
Legacy POPs				
Aldrin	X			A
Chlordanes^c^	X			A
Endrin	X			A
Dichlorodiphenyltrichloroethane (DDT)^d^	X			B
Dieldrin	X			A
Heptachlor	X			A
Hexachlorobenzene (HCB)	X	X	X	A,C
Mirex	X			A
Polychlorinated biphenyls (PCBs)		X	X	A,C
Polychlorinated dibenzo-*p*-dioxins (PCDDs)		X	X	C
Polychlorinated dibenzofurans (PCDFs)		X	X	C
Toxaphene	X			A
Newer POPs				
Alpha and beta hexachlorocyclohexanes (α-HCHs, β-HCH)	X			A
Chlordecone	X			A
Dechlorane Plus		X		A
Dicofol	X			A
Endosulfan	X			A
Hexabromobiphenyl (HBB)		X		A
Hexabromocyclododecane (HBCD)		X		A
Hexachlorobutadiene (HCBD)		X	X	A,C
Lindane	X			A
Methoxychlor	X			A
Pentachlorobenzene (PeCB)	X	X	X	A,C
Pentachlorophenol (PCP)		X		A
Perfluorohexane sulfonate (PFHxS)		X		A
Perfluorooctanoic acid (PFOA)		X		A
Perflourooctane sulfonate (PFOS)		X		B
Perfluorooctane sulfonyl fluoride (PFOSF)		X		B
Polybrominated diphenyl ethers (PBDEs)^e^		X		A
Polychlorinated naphthalenes (PCNs)		X	X	A,C
Short chained chlorinated paraffins (SCCPs)		X		A
UV-238		X		A

a Stockholm Convention website: Stockholm Convention POPs (Last accessed 10/02/2024).

b Annex A POPs to be eliminated from production and use with specific exemptions; Annex B= POPs to be restricted from production and use with specific exemptions/acceptable uses; Annex C= POPs formed and released unintentionally from anthropogenic sources.

c Includes mixture of chlordane isomers (e.g., *cis*-chlordane, *trans*-chlordane, *trans*-nonachlor, *cis*-nonachlor)

d Includes primary compound (*p,p*’-DDT), impurities (*o,p*’-DDT), and major degradates (*p,p*’-dichlorodiphenyldichloroethylene (DDE), *o,p*’-DDE, *p,p*’-dichlorodiphenyldichloroethane (DDD), *o,p*’-DDD).

e Includes tetrabromodiphenyl ether (tetraBDE), pentabromodiphenyl ether (pentaBDE), hexabromodiphenyl ether (hexaBDE) and heptabromodiphenyl ether (commercial mixture octaBDE), decabromodiphenyl ether, commercial mixture (c-decaBDE).

**Table 2 |:** Time series studies evaluating the influence of climate and ecological parameters on generally declining temporal trends of legacy POP bioaccumulation with use reductions in Arctic and subarctic marine populations (additional information in Supplementary Table 4)

Species and tissue	Region	Reported climate-related parameter	Reported ecological parameter	Chemicals	Influence on trend	Years	Ref.

Thick-billed murre (*U. lomvia*), eggs	Canadian Arctic	↑ice free season	Diet shifts to subarctic prey	∑PCBs, HCB, heptachlor, oxyCHL, *p,p*’-DDE	↓	1993–2013	115

Thick-billed murre (*U. lomvia*), eggs	Canadian Arctic	↑rainfall	Not evaluated	CHBs, *p,p*’-DDE, dieldrin, most PCBs	↑^a^	1975–2014	109

				oxyCHL	↓		

Northern fulmar (*F. glaciaris*), eggs	Canadian Arctic	↑NAO	Not evaluated	CHBs, *cis-*and *trans*-nonachlors, dieldrin, mirex	↑	1975–2014	109

Common Eider (*S. mollissima*), serum	Canadian Arctic, subarctic	Colder years	Nesting/fasting, lipid mobilization	PCB-153, *p,p*′-DDE, HCB	↑^a^	2005–2009	232

Glaucus gull (*L. hyperboreus*), serum	Norwegian Arctic	↑AO	Diet shifts, foraging location	∑PCBs, HCB, oxyCHL	↑	1997–2006	114

Black-legged kittiwake (*R. trydactila*), serum	Norwegian Arctic	Not evaluated	Nesting/fasting, chick rearing	PCB-153	↓	2007–2011	116

				HCB, *p,p*’-DDE	↑		

Goldeneye (*B. clangula*), eggs	Norway	↑winter NAO	Diet shifts	*p,p*’-DDT, α-HCHs, oxyCHL, less persistent PCBs, nonachlors	↓	1999–2019	207

				*p,p*’-DDE, persistent PCBs, PFAS, more persistent PCBs	↔		

				HCB, β-HCH	↑		

Ringed seals (*P. hispida*), blubber	Canadian Arctic	Earlier sea ice breakup (≥12 days from annual mean)	Not evaluated	*p,p*’*-*DDE, PCBs	↑	1993–2008	242

				HCHs, CHLs, dieldren, mirex	↔		

				HCB, *p,p*’-DDT	↓		

Ringed seals (*P. hispida*), blubber	Canadian Arctic	↑AO, NAO (preceding year), sea ice (regional variability)	Not evaluated	∑PCBs, ∑_10_PCBs, ∑CHLs, ∑HCH, ∑DDT, oxyCHL, nonachlor	↑^b^	1972–2016	243

Ringed seals (*P. hispida*), blubber	Western Greenland	↑winter AO, ↓sea ice cover, ↑salinity	Diet (muscle δ^15^N)	PCB-153 (↑winter AO, ↓sea ice); PCB-52, *p,p*’*-*DDE, β-HCH (↑salinity); HCB (δ^15^N)	↑	1994–2010	244

				α-HCH (↑winter AO)	↓		

Arctic char (*S. alpinus*), muscle; Ringed seals (*P. hispida*), blubber	Eastern/Western Greenland	↑air temp, ↑total sea ice (preceding year), ↑winter AO	Diet (muscle δ^15^N)	∑_10_PCB, PCB-52, PCB-153, ∑DDT, HCB (western seals)	↑	1986–2016	222

Beluga whales (*D. leucas*), blubber	Canadian Arctic	Not evaluated	Diet (liver δ^15^N)	∑PCBs, ∑PBDE, ∑CHLs, ∑DDT, HCHs	↔	1987–2007	117

Polar bear (*U. maritimus*), serum	US/Alaskan Arctic	Not evaluated	Diet shifts to onshore foraging	∑CHLs	↓	2007–2014	192

				∑DDT, ∑PCBs, OCPs, ∑HCHs	↔		

Polar bear (*U. maritimus*), serum	Canadian Arctic	Sea ice breakup	Diet shifts to subarctic seals	∑PCBs, ∑CHLs, ∑PBDEs, β-HCH	↑	1991–2007	219

				∑DDT	↓		

				α-HCH	↔		

Polar bear (*U. maritimus*), serum	Eastern Greenland	↑NAO	Diet shifts to subarctic seals	∑PCBs, oxyCHL, *trans*-nonachlor, some PCBs, ∑DDT, *p,p*’-DDE, ∑PBDEs	↔	1984–2011	220

Polar bear (*U. maritimus*), serum; Arctic fox (*V. lagopus*), liver	Norwegian Arctic	↑sea ice^c^	Diet shifts	PFCAs (C9-C13)	↑	2000–2014	245

				PFCAs (C6-C8)	↓		

				PFSAs (C6-C8)	↓^d^		

Arctic fox (*V. lagopus*), liver	Norwegian Arctic	↑sea ice	Diet shifts to marine foraging	∑PCBs, ∑PBDEs, ∑CHLs, *p,p*’*-*DDE, HCB, β-HCH	↑	1997–2013	246

a Spatiotemporal variation with greater apparent lipid mobilization of POPs in colder Arctic populations compared to subarctic populations.

b Overall declining trends with putative use reductions but positive correlations with ↑AO and NAO and ↑total sea ice and variations by region.

c Additional climate and ecological factors reported included potentially elevated PFAS in marine-feeding bears due to elevated energy needs with larger home ranges and migration of PFAS from sea ice to water with plankton blooms. However, changes in PFAS emissions outweighed the influence of feeding habits in bears.

d Overall declines in PFOS and PFHxS in bears and foxes occurred until 2009/2010 after which declines level off.

↑ Increasing trend, ↓ Decreasing trend, ↔ No statistically significant influence of climate or ecological factors on trend, *AO* Arctic Oscillation, *CHB* Chlorobenzenes, *CHL* Chlordane, *DDT* dichlorodiphenyltrichloroethane, DDD Dichlorodiphenyldichloroethane; *DDE* Dichlorodiphenyldichloroethylene, *HCB* Hexachlorobenzene, *HCH* Hexachlorocyclohexane, *nonachlor* CHL impurities include cis and trans isomers, *NAO* North Atlantic Oscillation, *PCBs* Polychlorinated biphenyls, Perfluoroalkyl carboxylic acid, *PFOS* Perfluorooctane sulfonic acid, *PFSA* Perfluoroalkyl sulfonic acid, *PFAS* Per- and polyfluoroalkyl substances.

**Table 3 |:** Time series studies evaluating the influence of climate and ecological parameters on temporal trends of legacy POP bioaccumulation in Antarctic marine populations (additional information in Supplementary Table 4)

Species and tissue	Region	Reported climate parameter	Reported ecological parameter	Chemicals	Influence on trend	Years	Ref.

Emerald rockcod *(T. bernacchii),* muscle, liver	Ross Sea	↑ Ross Ice Shelf calving, 2000–2003	Not evaluated	PCBs, *p,p*′-DDT, *p,p*′- DDE, HCB	↓	1981–2010	29

					↑	2001–2005	

Humped rockcod (*G. gibberifrons),* blackfin icefish (*C. aceratus),* liver	Elephant Island	Not evaluated	Trophic position/benthos feeder	PCB-153, nonachlors, toxaphene congener, *p,p*′-DDE, mirex	↑	1987–1996	91

Adelie penguin *(P. adeliae),* subcutaneous fat (collected corpses)	Palmer Archipelago; Cape Crozier, Ross Island	Not evaluated	Sea ice foraging behavior (no link to prey trophic level)	*p,p*′-DDT: *p,p*′-DDE ratio	↓	1964–2006	247

				∑DDT	↔↑^a^		

Adelie *(P. adeliae)* and Emperor (*A. forsteri)* penguins, snow petrel *(P. nivea),* Skua (*C. maccormicki),* eggs	Bransfield Strait	Not evaluated	Migration patterns	∑PCBs, ∑HCHs, ∑DDT	↔	1994–2005	248

Humpback whale (*M. novaeangliae),* blubber	Ross Sea, East Antarctica; Brainsfield Strait, Western Antarctica	↓sea ice	Feeding conditions	∑PCBs, ∑DDT, HCB	↑	2008–2013	206,221

a While ratios of *p,p*′-DDT to *p,p*′-DDE were reported to decline (suggesting older sources of DDT), in one penguin population (Western Antarctic Peninsula) the ∑DDT did not decline with

↑ *p,p*′-DDT suggesting an ongoing source (e.g., glacial meltwater, continuing use/emissions).

↑ Increasing trend, ↓ Decreasing trend, ↔ No statistically significant trend change and no apparent influence of climate or ecological factors when reported, *AO* Arctic Oscillation, CHL Chlordane, *DDT* dichlorodiphenyltrichloroethane, *DDD* Dichlorodiphenyldichloroethane, *DDE* Dichlorodiphenyldichloroethylene, *HCB* Hexachlorobenzene, *HCH* Hexachlorocyclohexane, nonachlors CHL impurity includes cis and trans isomers, *NAO* North Atlantic Oscillation, *PCBs* Polychlorinated biphenyls, *PFAS* Per- and polyfluoroalkyl substances.

**Table 4 |:** Summary of biological effects reported with dual exposures to climate change drivers and POP exposures relevant to marine biota (additional information in Supplementary Table 3)

Climate parameter	Endpoints evaluated	Principal receptor	Principal effects and POP co-exposure	Ref.

Increasing water temperature	Toxicokinetics	Teleosts: Rainbow trout (*O. mykiss*) Marine medaka (*O melastigma*)	• ↑accumulation (PCBs, DDT), ↑metabolism/↓half-life (PCBs), ↑bioactivation to OH-PCBs • ↑distribution PFOS, PFHxS to liver, brain, blood • Organ- and temperature-specific elimination t_1/2_ (PFOS, PFHxS); longest t_1/2_ at 7 °C in liver, 11 °C in brain, and 19 °C in kidney	125,129,133–135,152

		Amphibians: Leopard frog (*L. pipiens*)	• ↑uptake, elimination in tadpoles with ↑rearing temperature; null effects on steady-state tissue levels (PCBs, PBDEs)	123

		Invertebrates: Water flea (*D. magna*)	• ↑accumulation with increasing chain length (PFDoA > PFDA > PFOA)	137
	
	Toxicity	Teleosts: Silver perch (*B. bidyanus*) Rainbow trout (*O. mykiss*) Rainbow fish (*M. duboulayi*) Western carp gudgeon (*H. klunzingeri*) Zebrafish (*D. rerio*) Arctic char (*S. alpinus*) White seabream (*D. sargus*) European flounder (*P. flesus*)	• ↑mortality, multiple species; coldwater-acclimated species potentially more sensitive (endosulfan) • ↓time to effects (endosulfan) • ↑developmental effects, reduced survival (endosulfan; PCBs) • ↑anxiety responses (BDE-209) • ↓body size (∑PCBs, ∑PBDEs)	130–132,160,163,202

		Amphibians: Striped marsh frog (*L. peronii*)	• ↓tadpole body size, growth, ↑predation (endosulfan)	199

		Invertebrates: Hood coral (*S. pistillata*) Water flea (*D. magna*)	• ↓photosynthesis, ↑oxidative stress (coral, PFOS) • Synergistic effects on immobilization (*D. magna;* PFAS mixture of PFDoA, PFDA, PFOA)	137,158
	
	Thermal tolerances	Teleosts: Silver perch (*B. bidyanus*) Rainbow trout (*O. mykiss*) Rainbow fish (*M. duboulayi*) Western carp gudgeon (*H. klunzingeri*) Marine medaka (*O. melastigma*) European flounder (*P. flesus*),	• ↓cTmax temperature, multiple species; coldwater fish possibly more sensitive than warmwater fish (endosulfan) • ↓thermal acclimation capacity in POP exposed/adapted fish populations (∑PCBs, ∑PBDEs) • ↓thermal tolerance and stress responses, switch to anaerobic respiration (DDT)	130,131,152,160
	
	Bioenergetics	Teleosts: European flounder (*P. flesus*) White seabream (*D. sargus*)	• ↓lipid across tissues (PCB-153) • ↓lipid/altered energy allocation (BDE-209) • ↓mitochondrial energy metabolism in PCB/PBDE adapted populations • Altered lipid homeostasis, ↓burst speed (*p,p*’-DDE pesticide mix)	160,161,200

Increasing acidification	Toxicokinetics, endocrine effects	Teleosts: Atlantic cod (*G. morhua*)	• Hypercapnia and PFOS ↑sex steroids (17β-estradiol, testosterone, and 11-ketotestosterone)	173

		Mollusks: Mediterranean mussel (*M. galloprovincialis*), Manila clam (*R. philippinarum*)	• Acidification ↓bioaccumulation in bivalve (PFOA, PFOS); addition of warming negated ↓bioaccumulation of PFOS • Acidification ↑bioaccumulation of dechloranes in bivalve, including with warming added	169

		Echinoderms: Sea urchin (*P. lividus*)	• ↓sperm motility and fertilization success with acidification alone; null effects of PFOS (short 30 min exposure complicates interpretation)	170

Increasing salinity	Osmoregulation	Teleosts: Tilapia (*S. melanotheron*)	• Perturbed osmoregulatory responses in saltwater-adapted fish (DDT)	175

*BDE-209* Decabromodiphenyl ether, *cTmax* Critical thermal maximum, *DDE* dichlorodiphenyldichloroethylene, *DDT* Dichlorodiphenyltrichloroethane, *PBDE* Polybrominated diphenyl ether, *PCB* polychlorinated biphenyl, *PCB-153* Hexachlorobiphenyl, *PFDA* Perfluorodecanoic acid, *PFDoA* Perfluorododecanoic acid, *PFOA* Perfluorooctanoic acid, *PFHxS* Perfluorohexane sulfonate, *PFOS* Perfluorooctane sulfonate, t_1/2_ half-life.

## Data Availability

Data sharing is not applicable to this article as no datasets were generated or analysed during the current study.
