# Supplementary material for: Climate change drives persistent organic pollutant dynamics in marine environments
Source: Commun Earth Environ. Author manuscript; Available in PMC 2026 May 13. (PMC12973078; doi:10.1038/s43247-025-02348-4)
Supplement: Supplement [file NIHMS2082204-supplement-Supplement.pdf]

SUPPLEMENTARY INFORMATION

Climate drivers and persistent organic pollutants in marine environments: a systematic review of fate, exposure, and effects

Pamela D. Noyes[1*], Daniele Miranda[2], Gabriel Oliveira de Carvalho[3], Alessandra Perfetti-Bolaño[4], Yago Guida[3], Fábio Barbosa Machado Torres[3], João Paulo Machado Torres[3], Karina S.B. Miglioranza[5], Vanessa Hatje[6,7], and Ricardo O. Barra[4,8]

1. Integrated Climate Sciences Division, Center for Public Health and Environmental Assessment, Office of Research and Development, U.S. EPA, Washington, DC, USA
2. University of Notre Dame, Department of Biological Sciences, Notre Dame, IN, USA
3. Instituto de Biofísica Carlos Chagas Filho, Centro de Ciências da Saúde, Universidade Federal do Rio de Janeiro, Ilha do Fundão, 21941-902, Rio Janeiro, RJ, Brazil
4. Facultad de Ciencias Ambientales y Centro EULA, Centro FONDAP CRHIAM, Universidad de Concepción, 4070386, Concepción, Chile
5. Universidad Nacional de Mar del Plata, IIMyC (CONICET), Mar del Plata, Argentina
6. Centro Interdisciplinar de Energia e Ambiente (CIEnAm) & Dept of Analytical Chemistry, Universidade Federal da Bahia, Salvador, Brazil
7. Marine Environmental Studies Laboratory, IAEA Marine Environment Laboratories, Department of Nuclear Sciences and Applications, International Atomic Agency, Monaco, Principality of Monaco
8. Instituto Milenio de Socio-Ecología Costera (SECOS), Concepción, Chile

***Corresponding Author:** Pamela Noyes (email: noyes.pamela@epa.gov)

Content:

Table 1. Web of Science and Scopus search strategies.

Table 2. Governmental and non-governmental reports examining climate change impacts on the fate, transport, impacts, and effects of POP exposures in marine environments**.**

Table 3. Biological effect studies of marine species evaluating combined climate change-related processes and POP exposures.

Table 4. Time series studies in marine populations evaluating POP time trends with climate and/or ecological parameters.

Table 1. Web of Science and Scopus search strategies.

Search	Search terms	Date
Original search	**Web of Science:** [TS= (“climat* chang*” OR “global warming” OR “chang* climat*” OR “chang* weather” OR “warming weather” OR “environment* temperature*” OR “greenhouse effect*” OR “ocean* acidification” OR “ocean* deoxygenation” OR “ice* melt*” OR “snow melt*” OR “glaci* melt*”) AND TS= (“persistent organic pollutant*” OR “POPs” OR “POP” OR “organochlorin* pesticide*” OR “OCP” OR “OCPs” OR “aldrin” OR “endrin” OR “dieldrin” OR “endosulfan” OR “endosulfans” OR “chlordane” OR “chlordanes” OR “chlordecone” OR “DDT” OR “DDTs” OR “DDE” OR “DDEs” OR “DDD” OR “DDDs” OR “heptachlor” OR “mirex” OR “polychlorinated biphenyl” OR “polychlorinated biphenyls” OR “PCB” OR “PCBs” OR “toxaphene” OR “*dioxin*” OR “PCDD*” OR “polychlorinated dibenzofuran*” OR “PCDF” OR “PCDFs” OR “pentachlorobenzene” OR “PeCB” OR “*bromodiphenyl ether” OR “polybrominated diphenyl ether” OR “PBDE” OR “PBDEs” OR “hexabromocyclododecane” OR “HBCDD” OR “HBCD” OR “hexachlorobenzene” OR “HCB” OR “hexachlorobutadiene” OR “HCBD” OR “hexachlorocyclohexane” OR “HCH” OR “HCHs” OR “lindane” OR “polychlorinated naphthalene” OR “polychlorinated naphthalenes” OR “PCN” OR “PCNs” OR “pentachlorophenol” OR “PCP” OR “perfluorooctanoic acid” OR “PFOA” OR “short*chain chlorinated paraffin*” OR “SCCP” OR “SCCPs” OR “perfluorooctane sulfonic acid” OR “PFOS” OR “PFOSF” OR “perfluorooctane sulfonyl fluoride” OR "Hexabromobiphenyl" OR "HBB" OR "dicofol")]	No date limit to August 2022
Original search	**Scopus:** [TITLE-ABS-KEY= (“climat* chang*” OR “global warming” OR “chang* climat*” OR “chang* weather” OR “warming weather” OR “environment* temperature*” OR “greenhouse effect*” OR “ocean* acidification” OR “ocean* deoxygenation” OR “ice* melt*” OR “snow melt*” OR “glaci* melt*”) AND TITLE-ABS-KEY= (“persistent organic pollutant*” OR “POPs” OR “POP” OR “organochlorin* pesticide*” OR “OCP” OR “OCPs” OR “aldrin” OR “endrin” OR “dieldrin” OR “endosulfan” OR “endosulfans” OR “chlordane” OR “chlordanes” OR “chlordecone” OR “DDT” OR “DDTs” OR “DDE” OR “DDEs” OR “DDD” OR “DDDs” OR “heptachlor” OR “mirex” OR “polychlorinated biphenyl” OR “polychlorinated biphenyls” OR “PCB” OR “PCBs” OR “toxaphene” OR “*dioxin*” OR “PCDD*” OR “polychlorinated dibenzofuran*” OR “PCDF” OR “PCDFs” OR “pentachlorobenzene” OR “PeCB” OR “*bromodiphenyl ether” OR “polybrominated diphenyl ether” OR “PBDE” OR “PBDEs” OR “hexabromocyclododecane” OR “HBCDD” OR “HBCD” OR “hexachlorobenzene” OR “HCB” OR “hexachlorobutadiene” OR “HCBD” OR “hexachlorocyclohexane” OR “HCH” OR “HCHs” OR “lindane” OR “polychlorinated naphthalene” OR “polychlorinated naphthalenes” OR “PCN” OR “PCNs” OR	No date limit to August 2022

Search	Search terms	Date
	"pentachlorophenol" OR "PCP" OR "perfluorooctanoic acid" OR "PFOA" OR "short*chain chlorinated paraffin*" OR "SCCP" OR "SCCPs" OR "perfluorooctane sulfonic acid" OR "PFOS" OR "PFOSF" OR "perfluorooctane sulfonyl fluoride" OR "Hexabromobiphenyl" OR "HBB" OR "dicofol")]	
Literature update	**Web of Science:** [TS= ("climat* chang*" OR "global warming" OR "chang* climat*" OR "chang* weather" OR "warming weather" OR "environment* temperature*" OR "greenhouse effect*" OR "ocean* acidification" OR "ocean* deoxygenation" OR "ice* melt*" OR "snow melt*" OR "glaci* melt*") AND TS= ("persistent organic pollutant*" OR "POPs" OR "POP" OR "organochlorin* pesticide*" OR "OCP" OR "OCPs" OR "aldrin" OR "endrin" OR "dieldrin" OR "endosulfan" OR "endosulfans" OR "chlordane" OR "chlordanes" OR "chlordecone" OR "DDT" OR "DDTs" OR "DDE" OR "DDEs" OR "DDD" OR "DDDs" OR "heptachlor" OR "mirex" OR "polychlorinated biphenyl" OR "polychlorinated biphenyls" OR "PCB" OR "PCBs" OR "toxaphene" OR "*dioxin*" OR "PCDD*" OR "polychlorinated dibenzofuran*" OR "PCDF" OR "PCDFs" OR "pentachlorobenzene" OR "PeCB" OR "*bromodiphenyl ether" OR "polybrominated diphenyl ether" OR "PBDE" OR "PBDEs" OR "hexabromocyclododecane" OR "HBCDD" OR "HBCD" OR "hexachlorobenzene" OR "HCB" OR "hexachlorobutadiene" OR "HCBD" OR "hexachlorocyclohexane" OR "HCH" OR "HCHs" OR "lindane" OR "polychlorinated naphthalene" OR "polychlorinated naphthalenes" OR "PCN" OR "PCNs" OR "pentachlorophenol" OR "PCP" OR "perfluorooctanoic acid" OR "PFOA" OR "short*chain chlorinated paraffin*" OR "SCCP" OR "SCCPs" OR "perfluorooctane sulfonic acid" OR "PFOS" OR "PFOSF" OR "perfluorooctane sulfonyl fluoride" OR "Hexabromobiphenyl" OR "HBB" OR "dicofol")]	August 2022 to August 2024
Literature update	**Scopus:** [TITLE-ABS-KEY= ("climat* chang*" OR "global warming" OR "chang* climat*" OR "chang* weather" OR "warming weather" OR "environment* temperature*" OR "greenhouse effect*" OR "ocean* acidification" OR "ocean* deoxygenation" OR "ice* melt*" OR "snow melt*" OR "glaci* melt*") AND TITLE-ABS-KEY= ("persistent organic pollutant*" OR "POPs" OR "POP" OR "organochlorin* pesticide*" OR "OCP" OR "OCPs" OR "aldrin" OR "endrin" OR "dieldrin" OR "endosulfan" OR "endosulfans" OR "chlordane" OR "chlordanes" OR "chlordecone" OR "DDT" OR "DDTs" OR "DDE" OR "DDEs" OR "DDD" OR "DDDs" OR "heptachlor" OR "mirex" OR "polychlorinated biphenyl" OR "polychlorinated biphenyls" OR "PCB" OR "PCBs" OR "toxaphene" OR "*dioxin*" OR "PCDD*" OR "polychlorinated dibenzofuran*" OR "PCDF" OR "PCDFs" OR "pentachlorobenzene" OR "PeCB" OR "*bromodiphenyl ether" OR "polybrominated diphenyl ether" OR "PBDE" OR "PBDEs" OR "hexabromocyclododecane" OR	August 2022 to August 2024

Search	Search terms	Date
	“HBCDD” OR “HBCD” OR “hexachlorobenzene” OR “HCB” OR “hexachlorobutadiene” OR “HCBD” OR “hexachlorocyclohexane” OR “HCH” OR “HCHs” OR “lindane” OR “polychlorinated naphthalene” OR “polychlorinated naphthalenes” OR “PCN” OR “PCNs” OR “pentachlorophenol” OR “PCP” OR “perfluorooctanoic acid” OR “PFOA” OR “short*chain chlorinated paraffin*” OR “SCCP” OR “SCCPs” OR “perfluorooctane sulfonic acid” OR “PFOS” OR “PFOSF” OR “perfluorooctane sulfonyl fluoride” OR "Hexabromobiphenyl" OR "HBB" OR "dicofol")]	
Recent Stockholm Convention chemical additions of PFHxS, Dechlorane Plus, Methoxychlor, and UV-238	**Web of Science:** [TS=(“climat* chang*” OR “global warming” OR “chang* climat*” OR “chang* weather” OR “warming weather” OR “environment* temperature*” OR “greenhouse effect*” OR “ocean* acidification” OR “ocean* deoxygenation” OR “ice* melt*” OR “snow melt*” OR “glaci* melt*”) AND TS=("Dechlorane-plus" OR "DDC-CO" OR "UV-328" OR "uv326" OR "bdtd" OR "Methoxychlor" OR "dimethoxydiphenyltrichlorethane" OR "dmds" OR "PFHxS" OR "Perfluorohexane sulfonic acid" OR “Dechlorane” OR “dechlorane” OR “Perfluorohexane sulfonate” OR “Perfluorohexanesulfonic acid” OR “Perfluorohexanesulfonate” OR “perfluorohexansulfonate” OR “PFAS” OR “Per- and Polyfluoroalkyl Substances” OR "PFASs" OR "Poly- and perfluoroalkyl substances" OR "perfluorinated compounds")]<br><br>**Scopus:** [TITLE-ABS-KEY= (“climat* chang*” OR “global warming” OR “chang* climat*” OR “chang* weather” OR “warming weather” OR “environment* temperature*” OR “greenhouse effect*” OR “ocean* acidification” OR “ocean* deoxygenation” OR “ice* melt*” OR “snow melt*” OR “glaci* melt*”) AND TITLE-ABS-KEY= ("Dechlorane-plus" OR "DDC-CO" OR "UV-328" OR "uv326" OR "bdtd" OR "Methoxychlor" OR "dimethoxydiphenyltrichlorethane" OR "dmds" OR "PFHxS" OR "Perfluorohexane sulfonic acid" OR "Dechlorane" OR "dechlorane" OR "Perfluorohexane sulfonate" OR "Perfluorohexanesulfonic acid" OR "Perfluorohexanesulfonate" OR "perfluorohexansulfonate" OR "PFAS" OR "Per- and Polyfluoroalkyl Substances" OR "PFASs" OR "Poly- and perfluoroalkyl substances" OR "perfluorinated compounds")]	No date limit to September 2024

Table 2. Governmental and non-governmental reports examining climate change impacts on the fate, transport, impacts, and effects of POP exposures in marine environments.

Title	Year	Organization	Reference Link
Canadian Arctic Contaminant Assessment Report I	1997	NCP	https://pubs.aina.ucalgary.ca/ncp/41529.pdf
AMAP Assessment 2002: The Influence of Global Change on Contaminant Pathways to, within, and from the Arctic	2002	AMAP	https://www.amap.no/documents/doc/amap-assessment-2002-the-influence-of-global-change-on-contaminant-pathways-to-within-and-from-the-arctic/94
Canadian Arctic Contaminant Assessment Report II	2003	NCP	https://pubs.aina.ucalgary.ca/ncp/62497.pdf
Climate Change in the Context of Multiple Stressors and Resilience, Chapter 17 in Arctic Climate Impact Assessment Scientific Report	2005	ACIA	Arctic Climate Impact Assessment. Cambridge University Press, 1042p. https://www.amap.no/documents/download/1098/inline
Marine Pollution, Climate Change and the Resilience of Coastal Ecosystems	2006	UNEP	https://wedocs.unep.org/20.500.11822/7924
Climate Change and POPs: Predicting the Impacts	2011	UNEP/AMAP/GMP	https://www.amap.no/documents/doc/climate-change-and-pops-predicting-the-impacts/753
Combined Effects of Selected Pollutants and Climate Change in the Arctic Environment,	2011	AMAP	https://www.amap.no/documents/doc/combined-effects-of-selected-pollutants-and-climate-change-in-the-arctic-environment/747
Revised draft guidance on how to assess the possible impact of climate change on the work of the Persistent Organic Pollutants Review Committee	2013	UNEP	UNEP/POPS/POPRC.9/10 https://documents.un.org/access.nsf/get?OpenAgent&DS=UNEP/POPS/POPRC.9/INF/15&Lang=E
Canadian Arctic Contaminant Assessment Report III: Persistent organic pollutants in Canada's north	2013	NCP	https://science.gc.ca/site/science/en/northern-contaminants-program/publications/canadian-arctic-contaminants-assessment-report-series/canadian-arctic-contaminants-assessment-report-iii-2013-persistent-organic-pollutants-canadas-north
Arctic Health Risks: Impacts on health in the Arctic and Europe owing to climate-induced changes in contaminant cycling (ArkRisk)	2014	EC	https://cordis.europa.eu/project/id/226534/reporting; https://project.arcrisk.amap.no/
AMAP Assessment 2015: Temporal Trends in Persistent Organic Pollutants in the Arctic	2016	AMAP	https://www.amap.no/documents/doc/amap-assessment-2015-temporal-trends-in-persistent-organic-pollutants-in-the-arctic/1521
Chemicals of Emerging Arctic Concern	2017	AMAP	https://www.amap.no/documents/doc/amap-assessment-2016-chemicals-of-emerging-arctic-concern/1624
Influence of Climate Change on Transport, Levels, and Effects of	2016	AMAP	https://www.amap.no/documents/doc/influence-of-climate-change-on-transport-levels-

Title	Year	Organization	Reference Link
Contaminants in Northern Areas – Part 2			and-effects-of-contaminants-in-northern-areas-part-2/1561
AMAP Assessment 2016: Chemicals of Emerging Arctic Concern	2017	AMAP	https://www.amap.no/documents/doc/AMAP-Assessment-2016-Chemicals-of-Emerging-Arctic-Concern/1624
Adaptation Actions for a Changing Arctic: Perspectives from the Barents Area	2017	AMAP	https://www.amap.no/documents/doc/adaptation-actions-for-a-changing-arctic-perspectives-from-the-barents-area/1604
Adaptation Actions for a Changing Arctic: Perspectives from the Bering-Chukchi-Beaufort Region	2017	AMAP	https://www.amap.no/documents/doc/adaptation-actions-for-a-changing-arctic-perspectives-from-the-bering-chukchi-beaufort-region/1615
Contaminants in Canada's north: State of knowledge and regional highlights	2017	NCP	https://science.gc.ca/site/science/en/northern-contaminants-program/publications/canadian-arctic-contaminants-assessment-report-series/contaminants-canadas-north-state-knowledge-and-regional-highlights-2017
Human Health Assessment	2017	NCP	https://science.gc.ca/site/science/en/northern-contaminants-program/publications/canadian-arctic-contaminants-assessment-report-series/human-health-assessment-2017
Adaptation Actions for a Changing Arctic: Perspectives from the Baffin Bay/Davis Strait Region	2018	AMAP	https://www.amap.no/documents/doc/Adaptation-Actions-for-a-Changing-Arctic-Perspectives-from-the-Baffin-BayDavis-Strait-Region/1630
Biological Effects of Contaminants on Arctic Wildlife and Fish	2018	AMAP	https://www.amap.no/documents/doc/amap-assessment-2018-biological-effects-of-contaminants-on-arctic-wildlife-and-fish/1663
IPCC Special Report on the Ocean and Cryosphere in a Changing Climate	2019	IPCC	https://doi.org/10.1017/9781009157964.002
Chemicals, Wastes and Climate Change, Interlinkages and Potential for Coordinated Action	2021	BRS, MC, UNEP, UN	https://www.unep.org/resources/report/chemicals-wastes-and-climate-change-interlinkages-and-potential-coordinated-action
AMAP Assessment 2020: POPs and Chemicals of Emerging Concern: Influence of Climate Change	2021	AMAP	https://www.amap.no/documents/doc/amap-assessment-2020-pops-and-chemicals-of-emerging-arctic-concern-influence-of-climate-change/3580

ACIA= Arctic Climate Impact Assessment; AMAP= Arctic Monitoring Assessment Programme; BRS= Basel, Rotterdam and Stockholm Conventions; EC= European Commission; GMP= Global Monitoring Programme; MC= Minamata Convention on Mercury; NCP= Northern Contaminants Program; UNEP= United Nations Environment Programme; UN= United Nations

Table 3. Biological effect studies of marine species evaluating combined climate change-related processes and POP exposures.

POP tested	Study type, location, and sampling period	Species	Combined climate change drivers-POP responses	Reference
		Increasing water temperature		
BDE-209	56-day, dietary exposure (2% bw/d at 60 ng/g dw), 19°C, 24°C	White seabream (*D. sargus*), juveniles	• Altered energy budget allocations and ↓fat content	Anacleto et al. 2018[1]
BDE-209	56-day, dietary exposure, (2% bw/d at 60 ng/g dw), 19°C, 24°C, pCO2 = 500 and 1500 µatm	White seabream (*D. sargus*), juveniles	• ↑extended risk aversion (anxiety behaviors) in fish co-exposed to BDE-209 with ↑temperatures and/or acidification<br>• BDE-209 exposure alone (no climate drivers) appeared to induce opposing hyperactivity responses	Dias et al. 2023[2]
PFOS	28-day, aqueous exposure, 10, 100, 1000, and 4000 ng/L 25°C, 32°C	Scleractinian coral (*S. pistillata*)	• ↓photosynthetic efficiency (Fv/Fm) and net photosynthesis rates (Pnet)<br>• ↑oxidative stress biomarkers	Bednarz et al. 2022[3]
PCB-153	Seine Estuary, FR (highly polluted), Vilaine Estuary, FR (moderately polluted), and Mondego Bay, ESP (upper thermal edge), Sept-Oct 2009	European flounder (*P. flesus*), juveniles	• ↓liver lipids in Seine population compared to Vilaine and Mondego Bay; ↓muscle lipid in Seine and Mondego Bay populations compared to Vilaine<br>• Putative reduction in bioenergetic reserves in highly polluted and thermally stressed populations	Borcier et al. 2016[4]
Endosulfan	96-hr, aqueous exposure 14°C, 20°C	Striped marsh frog (*L. peronii*), tadpoles	• ↓growth, body length<br>• ↑predation	Broomhall 2004[5]
PCBs, PBDEs	Up to 13.6 weeks, 6-82 ng/g (PBDEs), 25-70 ng/g (PCB-70), 3.5-7 ng/g (PCB-126), 18-27°C	Leopard frog (*L. pipiens*), tadpoles	• No statistically significant effect on PCB-70, PCB-126, or PBDE (pentaPBDE commercial mixture DE-71) accumulation with↑rearing temperature had due to estimates of both ↑uptake and elimination<br>• Metabolism and toxicodynamics not evaluated.	Brown et al. 2021[6]
PCB mixtures (Aroclors 1248, 1254, 1260)	30-day, dietary exposure, 10 µg/g feed at 1.5% bw/d, 300-d depuration, 8-16°C	Rainbow trout (*O. mykiss*), juveniles	• ↓biological t½ of PCBs; ↑biotransformation of PCBs to OH-PCBs in fish exposed to PCB mixture	Buckman et al. 2007[7]
ΣPCBs	Mackenzie River Basin, Canada, 1988-2008	Burbot (*L. lota*), adults	• ↑bioavailability and bioaccumulation with ↑temperature	Carrie et al. 2010[8]

POP tested	Study type, location, and sampling period	Species	Combined climate change drivers-POP responses	Reference
DDT	Raleigh, NC, US (warm temperate); Hobart, TAS, AU (colder); Innisfail, QL, AU (tropical)	Fruit fly (*D. melanogaster*)	• Warm-temperate population most susceptible to DDT; Tropical population most resistant<br>• Cold-adapted population most phenotypic plasticity; Tropical population least genetic variation	Fournier-Level et al. 2016[9]
ΣPCBs, ΣPBDEs	Seine Estuary, FR (highly polluted), Vilaine Estuary, FR (moderately polluted), Aug 2006-Jan 2007	European flounder (*P. flesus*), juveniles	• ↓fish size with ↑temperature from highly polluted Seine but not from moderately polluted Vilaine<br>• ↓genes in mitochondrial energy metabolism in Seine fish<br>• ↑susceptibility to temperature stress in pollution-adapted Seine fish	Lavergne et al. 2015[10]
DDT	96-hour, aqueous, 0.002 and 0.1 µg/l, 15-32°C	Marine medaka (*O. melastigma*), larvae	• ↑uptake and bioaccumulation with ↑temperature<br>• ↓thermal tolerance compared to controls exposed to ↑temperatures alone<br>• Switch to anaerobic metabolism with DDT and ↑temperature<br>• ↓stress proteins (heat shock proteins) with DDT and ↑temperature	Li et al. 2022[11]
Endosulfan	14-day, aqueous, 0.3-1 µg/l (endosulfan); 5 mg/l (phenol)	Silver perch (*B. bidyanus*), Rainbow trout (*O. mykiss*), Rainbow fish (*M. duboulayi*), Western carp gudgeon (*H. klunzingeri*), juveniles	• ↓cTmax temperature in all species exposed to endosulfan	Patra et al. 2007[12]
Endosulfan	18-hr, aqueous exposure, 10 µg/l, 15-35°C	Silver perch (*B. bidyanus*), juveniles	• Null effects on gill ventilation frequency	Patra et al. 2009[13]
Endosulfan	96-hour, aqueous exposure, 0.8 - 1.8 µg/l, 15-35°C (5-25°C rainbow trout)	Silver perch (*B. bidyanus*), Rainbow trout (*O. mykiss*), Rainbow fish (*M. duboulayi*), Western carp gudgeon (*H. klunzingeri*), juveniles	• ↑toxicity (mortality) in perch<br>• ↓time to effect in rainbow trout<br>• potential ↑sensitivity of coldwater-acclimated species	Patra et al. 2015[14]
ΣPCBs, ΣDDT, ΣCHL, dieldren endosulfan, ΣHCB, heptachlor, methoxychlor	Santa Catarina, Brazil; 8 sampling sites	Mangrove oyster *(Crassostrea gasar)*	• Δ oxidative stress biomarkers with temperature and contaminants (interactive effects not evaluated)	Bastolla et al. 2024[15]

POP tested	Study type, location, and sampling period	Species	Combined climate change drivers-POP responses	Reference
Pesticide mixture (with *p,p'*-DDE)	Topeka, IL, USA (Hatchery)	Chinook salmon (*Oncorhynchus tshawytscha*), juveniles	• ↑temperature and pesticide mixture altered expression of genes involved in lipid homeostasis, and at higher temperatures reduced burst speed of juvenile chinook salmon<br>• Not possible to deduce the relative contribution of the *p,p'*-DDE exposure to the effects	Fuller et al. 2022[16]
Pesticide mixture (included *p,p'*-DDE)	Topeka, IL, USA (Hatchery)	Chinook salmon (*Oncorhynchus tshawytscha*), juveniles	• ↑temperature with pesticide mixture altered expression of neuroendocrine, dopaminergic, and olfactory genes, and impaired olfactory-related behavioral functioning<br>• Not possible to deduce relative contribution of *p,p'*-DDE exposure to effects because mixture tested	Fuller et al. 2022[16]; Magnuson et al. 2023[17]
∑PCBs (Aroclor 1244)	Intraperitoneal (500 ng/g, 1000 ng/g), 30-d pre-spawning, eggs/larvae at 4°C (control) and 8.5°C	Arctic char (*Salvelinus alpinus*), adult females, offspring	• ↓survival and ↑rate of yolk sac consumption (i.e., metabolism), ↑oxidative stress (SOD, CAT) and neurological (AChE) biomarkers, and ↑anxiolytic behaviors with ↑temperature	Réalis-Doyelle et al. 2023[18]
PFOS, PFHxS	Dietary exposure (500 ng/g, 42-d, at 7°C, 11°C, 19°C, and PBPK model	Rainbow trout (Oncorhynchus mykiss), PBPK model	• ↑liver, brain, and blood, and ↓muscle, with ↑temperature (suggesting ↑accumulation in highly perfused tissue with ↑cardiac output)<br>• Elimination $t_{1/2}$ was organ and temperature specific. Longest half-life was at 7°C in liver, 11°C in brain, and 19°C in kidney.	[19, 20]; Vidal et al. 2020;Vidal, 2020 #72}
Endosulfan	10, 50, 250 µg/L, post-fertilization to hatch; 1-hr thermal stress (28.5°C to 35°C)	Zebrafish (*Danio rerio*), embryonic	• ↑mortality and incomplete or severely impaired brain morphology with co-exposures than endosulfan or temperature stress alone.	Zaman et al. 2023[21]
PFOA, PFDA, PFDoA, ∑3PFAS	60, 120 µg/L (single PFAS), 120, 360 µg/L (∑3PFAS), 16°C (control), 24°C	Water flea (*Daphnia magna*)	• ↑uptake with ↑temperature; PFDoA>PFDA>PFOA<br>• Synergistic effects ↑temperature on immobilization with ∑3PFAS	Zhang et al. 2022[22]
		Increasing acidification		
Dechloranes, PFOA, PFOS	20-day, dietary 50 ng/g at 2% bw/d (Dec), aqueous 1 µg/l (PFOA/OS), 19°C or 23°C, pH 7.6 or pH 8	Marine/estuarine bivalves (*M. galloprovincialis, R. philippinarum*)	• ↓bioaccumulation of PFOA and PFOS with acidification; for PFOS, response eliminated with ↑temperature<br>• ↑bioaccumulation dechloranes with acidification, and for Dec-602 and Dec-603 when combined with ↑temperature	Maulvault et al. 2018[23]
PFOS	5-day (1 hr/d), aqueous, 100 and 200 µg/l, 9-day,	Atlantic cod (*G. morhua*), juveniles	• ↑muscle tissue levels of 17β-estradiol, testosterone, and 11-ketotestosterone (hypercapnia alone and with PFOS)	Preus-Olsen et al. 2014[24]

POP tested	Study type, location, and sampling period	Species	Combined climate change drivers-POP responses	Reference
	hypercapnia (0.3, 0.9% pCO2)		• Modest changes in metabolic and estrogen responsive genes	
PFOS	5 and 30 min (breeding), 0.5 µg/L PFOS, pH 8.1 and 7.7	Sea urchin (*Paracentrotus lividus*), males	• ↓sperm motility and fertilization success with acidification alone; no effect of PFOS but exposure too abbreviated for interpretation	Munari et al. 2022[25]
		Altered salinity		
DDT	12-day, aqueous, 0.1 g/l, fresh, seawater (35ppt), hypersalinity (70 ppt)	Tilapia (*S. melanotheron*), adults	• ↓ gill NKA activity and CFTR chloride channels in saltwater-adapted fish exposed to DDT	Riou et al. 2012[26]

Abbreviations: AChE= Acetylcholinesterase; BDE-209= Decabromodiphenyl ether; BMI= Body mass index; CAT= Catalase; CFTR= cystic fibrosis transmembrane conductance regulator; cTmax= Critical thermal maximum; CHL= Chlordane; DDD= Dichlorodiphenyldichloroethane; DDE= dichlorodiphenyldichloroethylene; DDT= Dichlorodiphenyltrichloroethane; HBCD- Hexabromcyclododecane; HCB= Hexachlorobenzene; PBPK = Physiologically-based pharmacokinetic (PBPK); PBDE= Polybrominated diphenyl ether; PCB= polychlorinated biphenyl; PCB-153= Hexachlorobiphenyl; PFDA= Perfluorodecanoic acid; PFDoA= Perfluorododecanoic acid; PFOA= Perfluorooctanoic acid; PFOS= Perfluorooctane sulfonic acid; PFSA= Perfluoroalkyl sulfonic acid; SOD= Superoxide dismutase; $t_{½}$ = half-life

Table 4. Time series studies in marine populations evaluating POP time trends with climate and/or ecological parameters.

POP tested	Study type, location, and sampling period	Species	Combined climate change drivers-POP responses	Reference
∑PCBs, ∑PBDEs, ∑CHLs, *p,p'*-DDE, HCB, β-HCH	Svalbard, Norway, 1997-2013	Arctic fox (*V. lagopus*), liver	• ↑ with shift to marine diets<br>• ↑liver levels in leaner foxes in poorer condition	Andersen et al. 2015[27]
ΣPCBs, OCPs, ΣHCHs, ΣCHLs, ΣDDT	Beaufort Sea, AK, USA 2007-2014	Polar bear (*U. maritimus*), adult, sub-adult	• ↓ΣCHL with behavioral/dietary shifts from sea ice prey (ringed seals) to onshore foraging (whale carcasses); null effects for other POPs<br>• ↓some zoonotic pathogens (*Brucella* spp, *T. gondii*) in onshore bears	Atwood et al. 2017[28]
ΣPCBs, ΣOCP	Beaufort Sea, AK, March-May, 2013 and 2014	Polar bear (*U. maritimus*), adult, sub-adult	• ↓BMI and offshore habitat use<br>• ↑PCBs resulted in ↓cytokine-index	Bourque et al. 2020 [29]
HBCD	Mesocosm study; coastal Baltic ecosystem, 13-day, 12.3 µg/l dietary	Phytoplankton, benthic bivalves (*L. balthica, C.*	• Variable effects on zooplankton and benthos	Bradshaw et al. 2017[30]

POP tested	Study type, location, and sampling period	Species	Combined climate change drivers-POP responses	Reference
	(phytoplankton), 4.5 ml/system	*glaucum*), *Hydrobiidae* mud snail		
∑PCBs, HCB, heptachlor, oxyCHL, *p,p′*-DDE	Prince Leopold Island, Nunavut, Canada, 1975-2011	Thick-billed murre (*U. lomvia*), eggs	• ↓POPs despite ↑length of ice free season and changes in trophic position of maternal prey (as measured by δ15N in eggs) in two colonies	Braune et al. 2015[31]
PCBs	Saglek Fjord and Lake Melville estuary, Labrador, Canada, 2008-2011	Ringed seal (*Pusa hispida*), sub-adults and adults, males and females	• Estuarine seal population had better feeding opportunities but was more vulnerable to sea ice loss and contaminants than Saglek Fjord seals with wider feeding ranges	Brown et al. 2023[32]
p,p′-DDT, -DDE, -DDD, PFOS, PCBs, HCHs, HCB, CHLs, PCBs, mirex	Jonsvatnet, Norway, 199-2019	Goldeneye duck (B. *clangula*), eggs	• ↓ p,p'-DDT, α-HCHs, oxyCHL, less persistent PCBs, nonachlors; ↑ HCB, β-HCH; ↔ *p,p′*-DDE, persistent PCBs , PFAS, more persistent PCBs<br>• OCPs positively associated with δ15N (↑with feeding at upper trophic levels)<br>• PFAS, CHLs, HCB generally positively associated with δ13N (↑marine diets vs freshwater diets)<br>• Positive association of winter NAO with PFOS in eggs, null with other POPs evaluated	Bustness et al. 2022[33]
PCB-153, HCB, *p,p′*-DDE	Svalbard, Norway, 2007-2011	Black-legged kittiwake (*R. trydactila*), serum	• ↓PCB-153 with egg laying (maternal transfer) across study years; ↑PCB-153 from prebreeding to chick rearing period<br>• ↑HCB across years and within breeding season from local sources; ↑*p,p′*-DDE with exposure in over-wintering grounds	Bustnes et al. 2017[34]
PCB-153, *p,p′*-DDE, HCB	Kongsfjorden, Svalbard (high Arctic), Grindøya (subarctic), Norway, 2005-2009	Common Eider (*S. mollissima*), serum (nesting)	• ↑all POPs during incubation fast (with use of lipid stores)<br>• Relative ↑POPs varied more in Arctic than subarctic eiders<br>• ↑PCB-153 in the subarctic colony<br>• ↑*p,p'*-DDE and HCB concentrations in Arctic colony late in incubation period<br>• Negative association of body mass, clutch size with POPs	Bustnes et al. 2012[35]
∑PCBs, HCB, oxyCHL	Bear Island, Norway, 1997-2006	Glaucus gull (*L. hyperboreus*), serum	• ↑POPs associated with ↑AO in the preceding summer and preceding winter<br>• AO current winter negatively associated with POP concentrations	Bustnes et al. 2010[36]
PFAS	Hudson Bay, Nunavut, Canada, 2016-2018	Thick-billed murres (*Uria lomvia*), breeding adults	• ↓body mass with ↑PFAS<br>• Altered thyroid hormone homeostasis and earlier hatch dates	Choy et al. 2022[37]

POP tested	Study type, location, and sampling period	Species	Combined climate change drivers-POP responses	Reference
PCBs, *p,p′*-DDT, *p,p′*-DDE, HCB	Southern Ocean, Antarctica, 1981-2005	Emerald rockcod (*T. bernacchii*), muscle, liver	• Modest general ↓ over time<br>• ↑from 2001-2005 linked to iceberg calving of Ross Ice Shelf, 2000-2003, and remobilization of POPs	Cincinelli et al. 2016[38], Corsolini et al. 2022[39]
∑PCBs, ∑HCHs, ∑DDT	Brainsfield Strait, Western Antarctica, 1994-2005	Adelie penguin (*P. adeliae*), Emperor penguin (*A. forsteri*) penguins, snow petrel (*P. nivea*), Skua (*C. maccormicki*), eggs	• POP levels in migrating colonies > sub-Antarctic > Antarctic non-migrating colonies but overall no change in trend	Corsolini et al. 2011[40]
PCBs, HCB, *p,p′*-DDE	Ross Sea, Antarctica, 1995-1996	Adelie penguin (*P. adeliae*), stomach contents	• Complete melting of sea ice off rookery, putative ↑in release of ice sequestered POPs<br>• Altered feeding behavior to sea ice loss in breeding penguins with shifts to fish (from krill) containing higher POP levels	Corsolini et al. 2003[41]
∑PCBs, ∑PBDEs, p,p′-DDE, HCB	Hudson Bay, Nunavut, Canada, 2016-2017	Thick-billed murres (*Uria lomvia*), breeding adults	• No associations circulating POPs to accelerated sea-ice break-up or thyroid hormone levels<br>• Sampling after breeding suggested transfer of POPs to eggs possibly influencing results	Esparza et al. 2022[42]
CHBs, *p,p′*-DDE, dieldrin, PCBs, heptachlor, oxyCHL, nonachlor	Prince Leopold Island, Nunavut, Canada, 1975-2014	Thick-billed murre (*U. lomvia*), Northern fulmar (*F. glaciaris*), eggs	• Overall ↓trend consistent with ↓use<br>• In murre eggs, ↑rainfall associated with ↑CHBs, *p,p′*-DDE, dieldrin, most PCBS; ↓CHLs, PCB-170, -180 (only oxyCHL had clear negative trend)<br>• In fulmar eggs, ↑NAO+ index associated with ↑CBs, cis-/trans-nonachlor, dieldrin, mirex (inverse with NAO- index)	Foster et al. 2019[43]
p,p′-DDT/DDE, CHLs, CHBs, PCBs, HCB,HCHs, dieldren, mirex	Ulukhaktok, NW, Canada, 1993-2008	Ringed seals (*P. hispida*), blubber	• Overall ↓trend of HCB, *p,p′*-DDT with ↓use<br>• ↑PCBs, *p,p′*-DDE in years with early ice breakup (≥12 days from annual mean); predicted related to ↑feeding time	Gaden et al. 2012[44]
POPs	New South Wales, Australia, 1998-2019	Long-nosed fur seal (*Arctocephalus forsteri*)	• ↑temporal trend of positive associations of liver concentrations of p,p′-DDE and PCB-153 with emaciated body condition	Hall et al. 2023[45]
PCBs	Barents Sea, Svalbard, Norway, 2007	Zooplankton and food webs	• ↑subarctic species and ↑PCB biomagnification in food webs with increasing subarctic species	Hallanger et al. 2011[46, 47, 48]

POP tested	Study type, location, and sampling period	Species	Combined climate change drivers-POP responses	Reference
∑PCBs, ∑10PCBs, ∑CHLs, ∑HCH, ∑DDT, oxyCHL, nonachlor	Multiple Canadian Arctic regions, 1972-2016	Ringed seals (*P. hispida*), blubber	• Overall ↓trend with ↓use<br>• Positive correlation of POPs with ↑AO and NAO and ↑total sea ice but with variations by region	Houde et al. 2019[49]
∑PCBs, ∑PBDE, ∑CHLs, ∑DDT, HCHs	St. Lawrence Estuary, Canada, 1987-2007	Beluga whale (D. leucas), blubber	• Modest ↓trend<br>• Null effects for role of trophic feeding level (as measured by liver δ15N)	Lebeuf et al. 2014[50]
α-HCH, β-HCH, ΣPCB, ΣCHL, ΣPBDE, ΣDDT	Western Hudson Bay, Canada, 1991-2007	Polar bear (*U. maritimus*), adult	• ↑trend of ΣPCBs and ΣCHL<br>• Faster ↑trend of β-HCH and ΣPBDEs<br>• Faster ↓trend of ΣDDT<br>• Dietary shifts from ice-associated bearded seal to open water harbor and harp seals; relatively constant consumption of ringed seal	McKinney et al. 2009[51]
PCBs, *p,p′*-DDE, dieldren, CHLs, heptachlor	Cumberland Sound, Nanavut, Canada Arctic, 2007-2008	Zooplankton, fish, marine mammal food webs	• ↑subarctic species and ↑biomagnification of tetrachlorinated - heptachlorinated PCBs*, p,p′*-DDE, dieldren, heptachlor epoxide (metabolite), trans-nonachlor (CHL constituent) in food webs with ↑subarctic species<br>• ↔ in biomagnification of trichlorinated PCBs, HCB, α-HCH	McKinney et al. 2012[52]
PCBs, HCB, α-HCH, β-HCH, ΣDDT, ΣCHL, PBDEs	Eastern coastal Greenland, 1984-2011	Polar bear (*U. maritimus*), adult	• Faster rate of ↑trend in brominated POPs; generally slower rate of downward trend of chlorinated POPs (both not statistically significant)<br>• Dietary shifts from ice associated, less contaminated ring seals to more contaminated hooded seals	McKinney et al. 2013[53]
ΣPCBs, ΣDDT, HCB	Moreton Bay, Australia and Antarctic feeding grounds, 2008-2013	Humpback whale (*M. novaeangliae*, adult males	• ↑POPs with climate-induced reductions in sea ice and poor feeding conditions in Antarctica foraging grounds	Nash et al. 2018[54]
ΣPCBs, ΣDDT, HCB	Moreton Bay, Australia, and Antarctic feeding grounds, 2017	Humpback whale (M. novaeangliae, adult males	• Anomalous climatic events in Antarctic in 2017 (lowest summer sea ice on record, large 10% cleave of Larsen C ice shelf, 300K m2 polynya in winter sea ice)<br>• Negative trends with five of six eco-physiological markers of population adiposity, fecundity, calf/juvenile survival, and a lag response for sixth marker, diet<br>• Adiposity third lowest across 12-year timeline and POP levels in outer blubber fourth highest across a 10-yr timeline	Nash et al. 2023[55]

POP tested	Study type, location, and sampling period	Species	Combined climate change drivers-POP responses	Reference
PCB, PAH, OCP, CHB mixture	Mesocosm study, coastal bacteria	Multiple species	• ↓bacterial abundance and diversity with ↑terrestrial DOM and POP mixture	Rodriguez et al. 2018[56]
∑**10**PCB, PCB-52, PCB-153, ∑DDT, HCB	Eastern and western Greenland, 1986-2016 and 1994-2016, respectively	Arcti char (*S. alpinus*), muscle<br>Ringed seal (*P. hispida*), adults, blubber	• ↓overall trends for all POPs except HCB that was stable in Arctic char and western seals<br>• ∑10PCB, PCB-153, α-HCH positively associated with ↑air temp<br>• ∑10PCB, PCB-52, PCB-153, ∑DDT, HCB positively associated with ↑extent of sea-ice in previous year in western seals<br>• ∑10PCB, PCB-52, PCB-153, ∑DDT positively associated with ↑water temp (preceding year) in western seals<br>• HCB, α-HCH positively associated with ↑winter AO and δ15N in eastern seals but AO inverse for α-HCH in western seals	Rigét et al. 2020[57]
PCB-52, PCB-153, *p,p′*-DDE, β-HCH, α-HCH	Disko Island, central west, Greenland, 1994-2010	Ringed seals (*P. hispida*), blubber	• Overall ↓POPs<br>• ↑PCB-153 and ↓α-HCH with ↑winter AO<br>• ↑PCB-52, *p,p′*-DDE, β-HCH with ↑salinity<br>• ↑HCB with ↑muscle (δ15N), higher trophic feeding	Rigét et al. 2013[58]
PFAS	Svalbard, Norway, 1997-2014	Polar bear (*U. maritimus*), serum; Arctic fox (*V. lagopus*), liver	• Dominant PFAS measured was PFOS followed by PFNA, PFHxS, and PFUnDA in bears and foxes<br>• ↑PFAS with ↑trophic feeding level and marine diet as inferred from ↑δ15N and δ13C, respectively<br>• Modest effect of altered climate variables linked to diet shifts on bioaccumulation	Routti et al. 2017[59]
PCBs, PBDEs, OCPs, HCHs, CHLs, ΣDDT, dieldrin	Eastern coastal Greenland, 1892-2010	Polar bear (*U. maritimus*), adult	• ↓trend of skull size and bone mineral density over time<br>• Equivocal findings for links to POPs and NAO index	Sonne et al. 2013[60]
PCBs, OCPs, PBDEs, CHLs, HCB, ΣDDT, HBCD	Barents Sea, Svalbard, Norway, April, Sept 2012 and 2013	Polar bear (*U. maritimus*), adult	• ↓body condition with sea ice loss (spatially and temporally); primary predictor of POP levels in plasma and adipose	Tartu et al. 2017[61]
PCBs, CHLs, PFCAs, PFSAs, OCPs, PBDEs, others	Barents Sea, Svalbard, Norway, April and Sept 2012, Sept 2013	Polar bear (*U. maritimus*), adult females	• General ↑lipid metabolism biomarkers that increased with sea ice loss	Tartu et al. 2017[62]

POP tested	Study type, location, and sampling period	Species	Combined climate change drivers-POP responses	Reference
PCBs, nonachlors, toxaphene congener, *p,p'*-DDE, mirex	Elephant Island, Southern Ocean, Antarctica, 1987-1996	Humped rockcod (*G. gibberifrons*), blackfin icefish (*C. aceratus*), mackerel icefish (*C. gunnari*), liver	• ↑PCB-153, nonachlors, toxaphene congener, *p,p'*-DDE, and mirex in the benthos feeder humped rockcod and fish feeder blackfin icefish in 1996 relative to 1987<br>• Generally null results in the krill feeder mackerel icefish for POPs evaluated<br>• Species differences suggest greater role for biomagnification than bioconcentration, and indication of potential ↑global distributions to Antarctic with climate change and ↑use in Southern Hemisphere	Weber and Goerke 2003[63]

Abbreviations: ↑= Increasing trend; ↓= Decreasing trend; ↔= No statistically significant trend change and no apparent influence of climate or ecological factors when reported; Alpha and beta hexachlorocyclohexanes= α-HCH, β-HCH; AO= Arctic Oscillation; CHBs= Chlorobenzenes; Chlordanes= CHL; Dichlorodiphenyltrichloroethane= DDT; dichlorodiphenyldichloroethylene= DDE; dichlorodiphenyldichloroethane= DDD; Hexachlorobenzene= HCB; NAO= North Atlantic Oscillation; OCP= Organochlorine pesticide; OCS= octachlorostyrene; PAH= Polycyclic aromatic hydrocarbon; PBDE= Polybrominated diphenyl ether; PCB= polychlorinated biphenyl; PCB-153= Hexachlorobiphenyl; PFCA= Perfluoroalkyl carboxylic acid; PFOS= Perfluorooctane sulfonic acid; PFSA= Perfluoroalkyl sulfonic acid

References

1. Anacleto P, Figueiredo C, Baptista M, Maulvault AL, Camacho C, Pousao-Ferreira P, Valente LMP, Marques A, Rosa R. Fish energy budget under ocean warming and flame retardant exposure. *Environmental Research* 164, 186-196 (2018). DOI:10.1016/j.envres.2018.02.023.
2. Dias M, Paula JR, Pousao-Ferreira P, Casal S, Cruz R, Cunha SC, Rosa R, Marques A, Anacleto P, Maulvault AL. Combined effects of climate change and BDE-209 dietary exposure on the behavioural response of the white seabream, *Diplodus sargus*. *Science of the Total Environment* 881, 163400 (2023). DOI:10.1016/j.scitotenv.2023.163400.
3. Bednarz VN, Choyke S, Marangoni LFB, Otto EI, Beraud E, Metian M, Tolosa I, Ferrier-Pages C. Acute exposure to perfluorooctane sulfonate exacerbates heat-induced oxidative stress in a tropical coral species. *Environmental Pollution* 302, 119054 (2022). DOI:10.1016/j.envpol.2022.119054.
4. Borcier E, Charrier G, Amerand A, Theron M, Loizeau V, Pedron N, Laroche J. Bioenergetic Transcriptomic Responses of European Flounder (Platichthys Flesus) Populations in Contrasted Environments: Impacts of Pollution and Global Warming. *Journal of xenobiotics* 6, 6586-6586 (2016). DOI:10.4081/xeno.2016.6586.
5. Broomhall SD. Egg temperature modifies predator avoidance and the effects of the insecticide endosulfan on tadpoles of an Australian frog. *Journal of Applied Ecology* 41, 105-113 (2004). DOI:10.1111/j.1365-2664.2004.00883.x.
6. Brown CT, Yahn JM, Karasov WH. Warmer temperature increases toxicokinetic elimination of PCBs and PBDEs in Northern leopard frog larvae (*Lithobates pipiens*). *Aquatic Toxicology* 234, 105806 (2021). DOI:10.1016/j.aquatox.2021.105806.
7. Buckman AH, Brown SB, Small J, Muir DCG, Parrott J, Solomon KR, Fisk AT. Role of temperature and enzyme induction in the biotransformation of polychlorinated biphenyls and bioformation of hydroxylated polychlorinated biphenyls by rainbow trout (*Oncorhynchus mykiss*). *Environmental Science & Technology* 41, 3856-3863 (2007). DOI:10.1021/es062437y.
8. Carrie J, Wang F, Sanei H, Macdonald RW, Outridge PM, Stern GA. Increasing contaminant burdens in an Arctic fish, burbot (*Lota lota*), in a warming climate. *Environmental Science & Technology* 44, 316-322 (2010). DOI:10.1021/es902582y.
9. Fournier-Level A, Neumann-Mondlak A, Good RT, Green LM, Schmidt JM, Robin C. Behavioural response to combined insecticide and temperature stress in natural populations of *Drosophila melanogaster*. *Journal of Evolutionary Biology* 29, 1030-1044 (2016). DOI:10.1111/jeb.12844.
10. Lavergne E, Pedron N, Calves I, Claireaux G, Mazurais D, Zambonino-Infante J, Le Bayon N, Cahu C, Laroche J. Does the chronic chemical contamination of a European flounder population decrease its thermal tolerance? *Marine Pollution Bulletin* 95, 658-664 (2015). DOI:10.1016/j.marpolbul.2015.01.006.
11. Li AJ, Zhou G-J, Lai RWS, Leung PTY, Wu CC, Zeng EY, Lui GCS, Leung KMY. Extreme cold or warm events can potentially exacerbate chemical toxicity to the marine medaka fish *Oryzias melastigma*. *Aquatic Toxicology* 249, 106226 (2022). DOI:10.1016/j.aquatox.2022.106226.
12. Patra RW, Chapman JC, Lim RP, Gehrke PC. The effects of three organic chemicals on the upper thermal tolerances of four freshwater fishes. *Environmental Toxicology and Chemistry* 26, 1454-1459 (2007). DOI:10.1897/06-156r1.1.
13. Patra RW, Chapman JC, Lim RP, Gehrke PC, Sunderam RM. Effects of temperature on ventilatory behavior of fish exposed to sublethal concentrations of endosulfan and chlorpyrifos. *Environmental Toxicology and Chemistry* 28, 2182-2190 (2009). DOI:10.1897/08-532.1.
14. Patra RW, Chapman JC, Lim RP, Gehrke PC, Sunderam RM. Interactions between water temperature and contaminant toxicity to freshwater fish. *Environmental Toxicology and Chemistry* 34, 1809-1817 (2015). DOI:10.1002/etc.2990.

15. Bastolla CLV, Guerreiro FC, Saldana-Serrano M, Gomes CHAM, Lima D, Rutkoski CF, Mattos JJ, Dias VHV, Righetti BPH, Ferreira CP, Martim J, Alves TC, Melo CMR, Marques MRF, Luechmann KH, Almeida EA, Bainy ACD. Emerging and legacy contaminants on the Brazilian southern coast (Santa Catarina): A multi-biomarker approach in oysters Crassostrea gasar (Adanson, 1757). *Science of the Total Environment* 925, (2024). DOI:10.1016/j.scitotenv.2024.171679.

16. Fuller N, Magnuson JT, Hartz KEH, Whitledge GW, Acuna S, McGruer V, Schlenk D, Lydy MJ. Dietary exposure to environmentally relevant pesticide mixtures impairs swimming performance and lipid homeostatic gene expression in Juvenile Chinook salmon at elevated water temperatures. *Environmental Pollution* 314, 120308 (2022). DOI:10.1016/j.envpol.2022.120308.

17. Magnuson JT, Fuller N, McGruer V, Acuna S, Hartz KEH, Whitledge GW, Lydy MJ, Schlenk D. Effect of temperature and dietary pesticide exposure on neuroendocrine and olfactory responses in juvenile Chinook salmon (*Oncorhynchus tshawytscha*). *Environmental Pollution* 318, 120938 (2023). DOI:10.1016/j.envpol.2022.120938.

18. Réalis-Doyelle E, Cottin N, Daufresne M, Naffrechoux E, Reynaud S, Guillard J. Evolution of pace-of-life syndrome under conditions of maternal PCB contamination and global warming in early life stages of cold stenothermic fish (Arctic char). *Aquatic Toxicology* 255, 106396 (2023). DOI:10.1016/j.aquatox.2023.106396.

19. Vidal A, Babut M, Garric J, Beaudouin R. Elucidating the fate of perfluorooctanoate sulfonate using a rainbow trout (*Oncorhynchus mykiss*) physiologically-based toxicokinetic model. *Science of the Total Environment* 691, 1297-1309 (2019). DOI:10.1016/j.scitotenv.2019.07.105.

20. Vidal A, Lafay F, Daniele G, Vulliet E, Rochard E, Garric J, Babut M. Does water temperature influence the distribution and elimination of perfluorinated substances in rainbow trout (*Oncorhynchus mykiss*)? *Environmental Science and Pollution Research* 26, 16355-16365 (2019). DOI:10.1007/s11356-019-05029-w.

21. Zaman T, Fahad TM, Rana M, Hossain MS, Mamun A, Haque MA, Sarker A, Islam MS, Haque MM-UL, Naz T, Manik MIN, Ali H, Yamasu K, Khan A. Endosulfan affects embryonic development synergistically under elevated ambient temperature. *Environmental Science and Pollution Research* 30, 73393-73404 (2023). DOI:10.1007/s11356-023-27665-z.

22. Zhang Q, Zhang Y, Hu D, Wen W, Xia X. An unexpected synergistic toxicity caused by competitive bioconcentration of perfluoroalkyl acid mixtures to Daphnia magna: Further promoted by elevated temperature. *Environmental Pollution* 315, 120336 (2022). DOI:10.1016/j.envpol.2022.120336.

23. Maulvault AL, Camacho C, Barbosa V, Alves R, Anacleto P, Fogaca F, Kwadijk C, Kotterman M, Cunha SC, Fernandes JO, Rasmussen RR, Sloth JJ, Aznar-Alemany O, Eljarrat E, Barcelo D, Marques A. Assessing the effects of seawater temperature and pH on the bioaccumulation of emerging chemical contaminants in marine bivalves. *Environmental Research* 161, 236-247 (2018). DOI:10.1016/j.envres.2017.11.017.

24. Preus-Olsen G, Olufsen MO, Pedersen SA, Letcher RJ, Arukwe A. Effects of elevated dissolved carbon dioxide and perfluorooctane sulfonic acid, given singly and in combination, on steroidogenic and biotransformation pathways of Atlantic cod. *Aquatic Toxicology* 155, 222-235 (2014). DOI:10.1016/j.aquatox.2014.06.017.

25. Munari M, Devigili A, dalle Palle G, Asnicar D, Pastore P, Badocco D, Marin MG. Ocean Acidification, but Not Environmental Contaminants, Affects Fertilization Success and Sperm Motility in the Sea Urchin *Paracentrotus lividus*. *Journal of Marine Science and Engineering* 10, 247 (2022).

26. Riou V, Ndiaye A, Budzinski H, Dugue R, Le Menach K, Combes Y, Bossus M, Durand J-D, Charmantier G, Lorin-Nebel C. Impact of environmental DDT concentrations on gill adaptation to increased salinity in the tilapia *Sarotherodon melanotheron*. *Comparative Biochemistry and Physiology C-Toxicology & Pharmacology* 156, 7-16 (2012). DOI:10.1016/j.cbpc.2012.03.002.

27. Andersen MS, Fuglie E, Konig M, Lipasti I, Pedersen AO, Polder A, Yoccoz NG, Routti H. Levels and temporal trends of persistent organic pollutants (POPs) in arctic foxes (*Vulpes lagopus*) from Svalbard in relation to dietary habits and food availability. *Science of the Total Environment* 511, 112-122 (2015). DOI:10.1016/j.scitotenv.2014.12.039.

28. Atwood TC, Duncan C, Patyk KA, Nol P, Rhyan J, McCollum M, McKinney MA, Ramey AM, Cerqueira-Cezar CK, Kwok OCH, Dubey JP, Hennager S. Environmental and behavioral changes may influence the exposure of an Arctic apex predator to pathogens and contaminants. *Scientific Reports* 7, 13193 (2017). DOI:10.1038/s41598-017-13496-9.

29. Bourque J, Desforges J-P, Levin M, Atwood TC, Sonne C, Dietz R, Jensen TH, Curry E, McKinney MA. Climate-associated drivers of plasma cytokines and contaminant concentrations in Beaufort Sea polar bears (*Ursus maritimus*). *Science of the Total Environment* 745, 140978 (2020). DOI:10.1016/j.scitotenv.2020.140978.

30. Bradshaw C, Golz A-L, Gustafsson K. Coastal Ecosystem Effects of Increased Summer Temperature and Contamination by the Flame Retardant HBCDD. *Journal of Marine Science and Engineering* 5, (2017). DOI:10.3390/jmse5020018.

31. Braune BM, Gaston AJ, Hobson KA, Gilchrist HG, Mallory ML. Changes in trophic position affect rates of contaminant decline at two seabird colonies in the Canadian Arctic. *Ecotoxicology and Environmental Safety* 115, 7-13 (2015). DOI:10.1016/j.ecoenv.2015.01.027.

32. Brown TM, Ogloff WR, Yurkowski DJ, Coffey J, Stenson G, Sjare B. Divergent habitat use and the influence of sea ice concentration on the movement behaviour of ringed seals *Pusa hispida* in Labrador, Canada. *Marine Ecology Progress Series* 710, 137-153 (2023). DOI:10.3354/meps14280.

33. Bustnes JO, Bardsen B-J, Herzke D, Bangjord G, Bollinger E, Bourgeon S, Schulz R, Fritsch C, Eulaers I. The impact of climate sensitive factors on the exposure to organohalogenated contaminants in an aquatic bird exploiting both marine and freshwater habitats. *Science of the Total Environment* 850, 157667 (2022). DOI:10.1016/j.scitotenv.2022.157667.

34. Bustnes JO, Bardsen B-J, Moe B, Herzke D, Hanssen SA, Sagerup K, Bech C, Nordstad T, Chastel O, Tartu S, Gabrielsen GW. Temporal variation in circulating concentrations of organochlorine pollutants in a pelagic seabird breeding in the high Arctic. *Environmental Toxicology and Chemistry* 36, 442-448 (2017). DOI:10.1002/etc.3560.

35. Bustnes JO, Moe B, Hanssen SA, Herzke D, Fenstad AA, Nordstad T, Borga K, Gabrielsen GW. Temporal Dynamics of Circulating Persistent Organic Pollutants in a Fasting Seabird under Different Environmental Conditions. *Environmental Science & Technology* 46, 10287-10294 (2012). DOI:10.1021/es301746j.

36. Bustnes JO, Gabrielsen GW, Verreault J. Climate Variability and Temporal Trends of Persistent Organic Pollutants in the Arctic: A Study of Glaucous Gulls. *Environmental Science & Technology* 44, 3155-3161 (2010). DOI:10.1021/es9032919.

37. Choy ES, Elliott KH, Esparza I, Patterson A, Letcher RJ, Fernie KJ. Potential disruption of thyroid hormones by perfluoroalkyl acids in an Arctic seabird during reproduction. *Environmental Pollution* 305, 119181 (2022). DOI:10.1016/j.envpol.2022.119181.

38. Cincinelli A, Martellini T, Pozo K, Kukucka P, Audy O, Corsolini S. <i>Trematomus bernacchii</i> as an indicator of POP temporal trend in the Antarctic seawaters. *Environmental Pollution* 217, 19-25 (2016). DOI:10.1016/j.envpol.2015.12.057.

39. Corsolini S, Ademollo N. POPs in Antarctic ecosystems: is climate change affecting their temporal trends? *Environmental Science-Processes & Impacts* 24, 1631-1642 (2022). DOI:10.1039/d2em00273f.

40. Corsolini S, Borghesi N, Ademollo N, Focardi S. Chlorinated biphenyls and pesticides in migrating and resident seabirds from East and West Antarctica. *Environment International* 37, 1329-1335 (2011). DOI:10.1016/j.envint.2011.05.017.

41. Corsolini S, Ademollo N, Romeo T, Olmastroni S, Focardi S. Persistent organic pollutants in some species of a Ross Sea pelagic trophic web. *Antarctic Science* 15, 95-104 (2003). DOI:10.1017/s0954102003001093.

42. Esparza I, Elliott KH, Choy ES, Braune BM, Letcher RJ, Patterson A, Fernie KJ. Mercury, legacy and emerging POPs, and endocrine-behavioural linkages: Implications of Arctic change in a diving seabird. *Environmental research* 212, 113190-113190 (2022). DOI:10.1016/j.envres.2022.113190.

43. Foster KL, Braune BM, Gaston AJ, Mallory ML. Climate Influence on Legacy Organochlorine Pollutants in Arctic Seabirds. *Environmental Science & Technology* 53, 2518-2528 (2019). DOI:10.1021/acs.est.8b07106.

44. Gaden A, Ferguson SH, Harwood L, Melling H, Alikamik J, Stern GA. Western Canadian Arctic Ringed Seal Organic Contaminant Trends in Relation to Sea Ice Break-Up. *Environmental Science & Technology* 46, 4427-4433 (2012). DOI:10.1021/es204127j.

45. Hall J, Nash SB, Gautam A, Bender H, Pitcher BJ, McCallum H, Doyle C. Persistent organic pollutants and trace elements detected in New Zealand fur seals (long-nosed fur seal; *Arctocephalus forsteri*) from New South Wales, Australia, between 1998 and 2019. *Science of the Total Environment* 902, 166087 (2023). DOI:10.1016/j.scitotenv.2023.166087.

46. Hallanger IG, Ruus A, Herzke D, Warner NA, Evenset A, Heimstad ES, Gabrielsen GW, Borga K. Influence of season, location, and feeding strategy on bioaccumulation of halogenated organic contaminants in arctic marine zooplankton. *Environmental Toxicology and Chemistry* 30, 77-87 (2011). DOI:10.1002/etc.362.

47. Hallanger IG, Ruus A, Warner NA, Herzke D, Evenset A, Schoyen M, Gabrielsen GW, Borga K. Differences between Arctic and Atlantic fjord systems on bioaccumulation of persistent organic pollutants in zooplankton from Svalbard. *Science of the Total Environment* 409, 2783-2795 (2011). DOI:10.1016/j.scitotenv.2011.03.015.

48. Hallanger IG, Warner NA, Ruus A, Evenset A, Christensen G, Herzke D, Gabrielsen GW, Borga K. Seasonality in contaminant accumulation in arctic marine pelagic food webs using trophic magnification factor as a measure of bioaccumulation. *Environmental Toxicology and Chemistry* 30, 1026-1035 (2011). DOI:10.1002/etc.488.

49. Houde M, Wang X, Colson TLL, Gagnon P, Ferguson SH, Ikonomou MG, Dubetz C, Addison RF, Muir DCG. Trends of persistent organic pollutants in ringed seals (<i>Phoca hispida</i>) from the Canadian Arctic. *Science of the Total Environment* 665, 1135-1146 (2019). DOI:10.1016/j.scitotenv.2019.02.138.

50. Lebeuf M, Measures L, Noel M, Raach M, Trottier S. A twenty-one year temporal trend of persistent organic pollutants in St. Lawrence Estuary beluga, Canada. *Science of the Total Environment* 485, 377-386 (2014). DOI:10.1016/j.scitotenv.2014.03.097.

51. McKinney MA, Peacock E, Letcher RJ. Sea Ice-associated Diet Change Increases the Levels of Chlorinated and Brominated Contaminants in Polar Bears. *Environmental Science & Technology* 43, 4334-4339 (2009). DOI:10.1021/es900471g.

52. McKinney MA, McMeans BC, Tomy GT, Rosenberg B, Ferguson SH, Morris A, Muir DCG, Fisk AT. Trophic Transfer of Contaminants in a Changing Arctic Marine Food Web: Cumberland Sound, Nunavut, Canada. *Environmental Science & Technology* 46, 9914-9922 (2012). DOI:10.1021/es302761p.

53. McKinney MA, Iverson SJ, Fisk AT, Sonne C, Riget FF, Letcher RJ, Arts MT, Born EW, Rosing-Asvid A, Dietz R. Global change effects on the long-term feeding ecology and contaminant exposures of East Greenland polar bears. *Global Change Biology* 19, 2360-2372 (2013). DOI:10.1111/gcb.12241.

54. Nash SMB, Castrillon J, Eisenmann P, Fry B, Shuker JD, Cropp RA, Dawson A, Bignert A, Bohlin-Nizzetto P, Waugh CA, Polkinghorne BJ, Dalle Luche G, McLagan D. Signals from the south; humpback whales carry messages of Antarctic sea-ice ecosystem variability. *Global Change Biology* 24, 1500-1510 (2018). DOI:10.1111/gcb.14035.

55. Nash SMB, Gross J, Castrillon J, Casa MV, Luche GD, Meager J, Ghosh R, Eggebo J, Nizzetto PB. Antarctic sea-ice low resonates in the ecophysiology of humpback whales. *Science of the Total Environment* 887, 164053 (2023). DOI:10.1016/j.scitotenv.2023.164053.

56. Rodríguez J, Gallampois CMJ, Timonen S, Andersson A, Sinkko H, Haglund P, Berglund ÅMM, Ripszam M, Figueroa D, Tysklind M, Rowe O. Effects of Organic Pollutants on Bacterial Communities Under Future Climate Change Scenarios. *Frontiers in Microbiology* 9, 2926 (2018). DOI:10.3389/fmicb.2018.02926.

57. Rigét F, Vorkamp K, Eulaers I, Dietz R. Influence of climate and biological variables on temporal trends of persistent organic pollutants in Arctic char and ringed seals from Greenland. *Environmental Science-Processes & Impacts* 22, 993-1005 (2020). DOI:10.1039/c9em00561g.

58. Rigét F, Vorkamp K, Hobson KA, Muir DCG, Dietz R. Temporal trends of selected POPs and the potential influence of climate variability in a Greenland ringed seal population. *Environmental Science-Processes & Impacts* 15, 1706-1716 (2013). DOI:10.1039/c3em00189j.

59. Routti H, Aars J, Fuglei E, Hanssen L, Lone K, Polder A, Pedersen AO, Tartu S, Welker JM, Yoccoz NG. Emission Changes Dwarf the Influence of Feeding Habits on Temporal Trends of Per- and Polyfluoroalkyl Substances in Two Arctic Top Predators. *Environmental Science & Technology* 51, 11996-12006 (2017). DOI:10.1021/acs.est.7b03585.

60. Sonne C, Bechshoft TO, Riget FF, Baagoe HJ, Hedayat A, Andersen M, Bech-Jensen J-E, Hyldstrup L, Letcher RJ, Dietz R. Size and density of East Greenland polar bear (*Ursus maritimus*) skulls: Valuable bio-indicators of environmental changes? *Ecological Indicators* 34, 290-295 (2013). DOI:10.1016/j.ecolind.2013.04.015.

61. Tartu S, Lille-Langoy R, Storseth TR, Bourgeon S, Brunsvik A, Aars J, Goksoyr A, Jenssen BM, Polder A, Thiemann GW, Torget V, Routti H. Multiple-stressor effects in an apex predator: combined influence of pollutants and sea ice decline on lipid metabolism in polar bears. *Scientific Reports* 7, 16487 (2017). DOI:10.1038/s41598-017-16820-5.

62. Tartu S, Bourgeon S, Aars J, Andersen M, Polder A, Thiemann GW, Welker JM, Routti H. Sea ice-associated decline in body condition leads to increased concentrations of lipophilic pollutants in polar bears (*Ursus maritimus*) from Svalbard, Norway. *Science of the Total Environment* 576, 409-419 (2017). DOI:10.1016/j.scitotenv.2016.10.132.

63. Weber K, Goerke H. Persistent organic pollutants (POPs) in antarctic fish: levels, patterns, changes. *Chemosphere* 53, 667-678 (2003). DOI:10.1016/s0045-6535(03)00551-4.
